# Vitamin in the Crosshairs: Targeting Pantothenate and Coenzyme A Biosynthesis for New Antituberculosis Agents

**DOI:** 10.3389/fcimb.2020.605662

**Published:** 2020-12-15

**Authors:** Hailey S. Butman, Timothy J. Kotzé, Cynthia S. Dowd, Erick Strauss

**Affiliations:** ^1^ Department of Chemistry, George Washington University, Washington, DC, United States; ^2^ Department of Biochemistry, Stellenbosch University, Stellenbosch, South Africa

**Keywords:** tuberculosis, coenzyme A, drug discovery, pantothenate, antimetabolite, drug resistance

## Abstract

Despite decades of dedicated research, there remains a dire need for new drugs against tuberculosis (TB). Current therapies are generations old and problematic. Resistance to these existing therapies results in an ever-increasing burden of patients with disease that is difficult or impossible to treat. Novel chemical entities with new mechanisms of action are therefore earnestly required. The biosynthesis of coenzyme A (CoA) has long been known to be essential in *Mycobacterium tuberculosis* (Mtb), the causative agent of TB. The pathway has been genetically validated by seminal studies *in vitro* and *in vivo*. In Mtb, the CoA biosynthetic pathway is comprised of nine enzymes: four to synthesize pantothenate (Pan) from l-aspartate and α-ketoisovalerate; five to synthesize CoA from Pan and pantetheine (PantSH). This review gathers literature reports on the structure/mechanism, inhibitors, and vulnerability of each enzyme in the CoA pathway. In addition to traditional inhibition of a single enzyme, the CoA pathway offers an antimetabolite strategy as a promising alternative. In this review, we provide our assessment of what appear to be the best targets, and, thus, which CoA pathway enzymes present the best opportunities for antitubercular drug discovery moving forward.

## Introduction

Despite a dramatic increase in innovative research over the last two decades, there remains a clear and pressing need for new antitubercular drugs. Annually, 1.4 million people continue to die of tuberculosis (TB), and the rates of drug resistant disease are on the rise “WHO MDR-TB Factsheet 2018” 2019; (World Health Organization, 2020). Caused by *Mycobacterium tuberculosis* (Mtb), drug-susceptible TB is typically treated using a cocktail of drugs, including rifampicin and isoniazid (World Health Organization, 2017). Mtb strains resistant to one or both of these two key TB drugs are exceedingly difficult to treat. As resistance to current drugs is a clear issue, compounds with mechanisms of action distinct from those of existing therapies are desperately needed.

### Why Target Pantothenate and Coenzyme A Biosynthesis in Mtb?

Cofactor biosynthetic pathways have drawn attention as potential targets for antimicrobial drug development since the demonstration that the sulfonamide class of antibacterials targets the dihydropteroate synthase (DHPS) enzyme involved in the biosynthesis of the B vitamin folate (Hammoudeh et al., 2013). This interest hinges on two important factors: first, cofactors are indispensable components of a wide range of metabolic reactions that sustain life, and second, the biosynthesis of cofactors—usually by biotransformation of a vitamin precursor—often shows sufficient differences in the manner in which it occurs in the pathogen compared to the human host that selective targeting presents itself as an achievable goal. In addition, most bacterial pathogens can produce the vitamin precursors through *de novo* synthesis, while humans must obtain them from their diet. This difference adds another layer of potential selective pressure that may also prove useful in the pursuit of cofactor-targeting antimicrobials.

It is therefore unsurprising that the biosynthesis and utilization of the enzyme cofactor coenzyme A (CoA, **1**) and its vitamin B_5_ precursor pantothenate (Pan, **2**) have a long history as targets in antimicrobial drug research, with the first studies following very close on the discovery of the sulfonamide antibacterials (Spry et al., 2008; Moolman et al., 2014; Wellington and Hung, 2018). As an enzyme cofactor, its use is unparalleled, with an estimation that up to 9% of all enzyme activities use CoA in one of its forms (Strauss, 2010). The most important of these are the enzymes involved in central carbon metabolism (itself an important potential target in Mtb (Rhee et al., 2011), and the large number of enzymes that use CoA as the acyl carrier for transfer of acyl groups in fatty acid and polyketide biosynthesis (Mercer and Burkart, 2007), as well as in signaling and regulation pathways relying on acetylation-based switches (Pietrocola et al., 2015).

**Figure 1:**
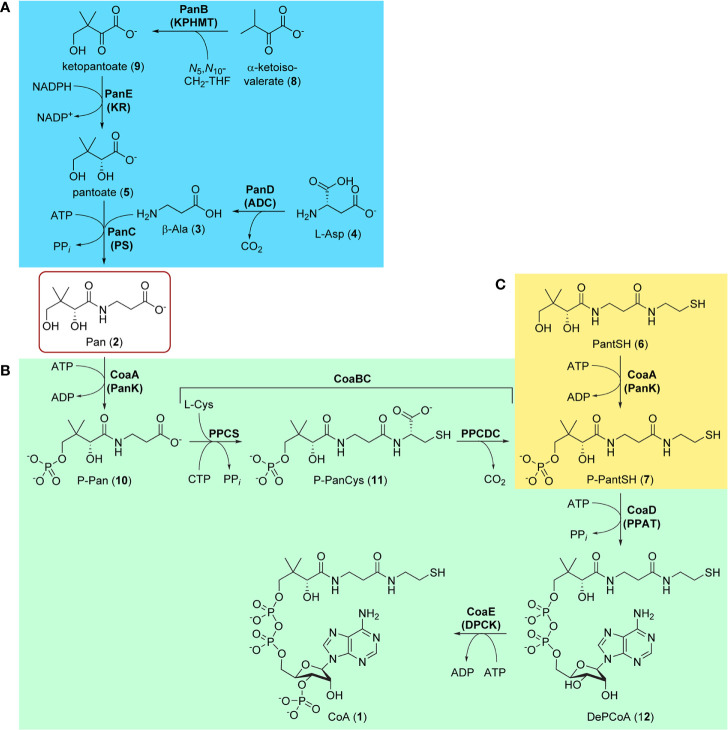
Biosynthesis of pantothenate (Pan, 1) and coenzyme A (CoA, 1). **(A)** Biosynthesis of Pan 2 proceeds in four steps (blue block): the decarboxylation of l-aspartate 4 by aspartate decarboxylase (PanD/ADC) to give β-Ala 3, the hydroxymethylation of α-ketoisovalerate (3-methyl-2-oxobutanoate, 8) by ketopantoate hydroxymethyltransferase (PanB/KPHMT) to form ketopantoate 9, and subsequent reduction of 9 by ketopantoate reductase (PanE/KR) to yield pantoate 5. In the final step, β-Ala 3 and pantoate 5 are coupled by pantothenate synthetase (PanC/PS) using ATP as intermediate activator to produce Pan 2. **(B)** CoA 1 is formed from Pan 2 in five steps (green block), the first being the ATP-mediated phosphorylation of Pan by pantothenate kinase (CoaA/PanK) to form 4′-phosphopantothenate (P-Pan, 10). P-Pan is then coupled to l-cysteine with the aid of cytidine triphosphate (CTP) as activator to form 4′-phosphopantothenoylcysteine (P-PanCys, 11), which is then decarboxylated to give 4′-phosphopantetheine (P-PantSH, 7). The transformation of P-Pan into P-PantSH is achieved by the PPCS and PPCDC enzymes, which are found on the bifunctional CoaBC protein. Adenylylation by phosphopantetheine adenylyltransferase (CoaD/PPAT) to form dephospho-coenzyme A (DePCoA, 12) and its ATP-mediated phosphorylation by dephospho-coenzyme A kinase (CoaE/DPCK) completes the pathway. **(C)** P-PantSH 7 can also be formed directly by PanK acting on the CoA degradation product pantetheine (PantSH, 6) as an alternative substrate (yellow block). This allows for the salvage biosynthesis of CoA in three steps using only PanK, PPAT, and DPCK.

CoA and Pan first drew attention in antituberculosis (antiTB) drug research with the report of a study of viable vaccine candidates that an Mtb mutant auxotrophic for Pan—due to the double deletion of the *panC* and *panD* genes involved in Pan biosynthesis—showed significantly lower virulence than the wild type strain, and that mice infected with this strain lived significantly longer (Sambandamurthy et al., 2002). This finding strongly indicated that in the host–pathogen interaction, Mtb is dependent on its own biosynthesis of Pan for optimal fitness, and also identified two enzymes that could potentially be targeted to achieve this through pharmacological intervention. The two enzymes in question, aspartate decarboxylase (ADC or PanD) and pantothenate synthase (PS or PanC), are responsible for the last steps of Pan biosynthesis, forming β-alanine **3** from l-aspartate **4** and coupling it to pantoate **5** (**Figure 1A**) (Strauss, 2010). The latter is a unique hydroxy acid formed by sequential action of ketopantoate hydroxymethyl transferase (KPHMT or PanB) and ketopantoate reductase (KR or PanE). This discovery sparked a large drug discovery effort aimed at finding inhibitors of PanC, and led to further interest in Pan and CoA biosynthesis as viable antiTB drug targets.

### The CoA Biosynthetic Pathway Is Essential in Mtb—But Does This Mean the Enzymes Are Good Drug Targets?

Like in other bacteria, the biosynthesis of CoA from Pan in Mtb occurs in five enzymatic steps catalyzed by four proteins, with one of these being bifunctional (**Figure 1B**) (Strauss, 2010). The pathway starts and ends with ATP-mediated phosphorylation reactions catalyzed by pantothenate kinase (PanK or CoaA) and dephospho-coenzyme A kinase (DPCK or CoaE). These reactions bracket the introduction of the key thiol group used in the acyl group carrying reactions through the coupling and subsequent decarboxylation of l-cysteine by phosphopantothenoylcysteine synthetase (PPCS) and decarboxylase (PPCDC) respectively, two enzyme activities harbored by the bifunctional CoaBC protein. The adenylyl group is introduced by phosphopantetheine adenylyltransferase (PPAT or CoaD). All of these enzymes are required for the *de novo* biosynthesis of CoA from Pan and have been shown to be essential (Sassetti et al., 2003; Ambady et al., 2012; DeJesus et al., 2017). Moreover, as no evidence exists that intact CoA can be obtained from the host, each enzyme presents itself *a priori* as a potential target for antiTB drug development.

However, recent findings in CoA biology have shown that CoA may in fact be obtained in other ways—although the physiological relevance of these alternative pathways is entirely dependent on the organism and its environmental context (Sibon and Strauss, 2016). For example, a salvage pathway exists in which the CoA breakdown product pantetheine (PantSH, **6**) can serve as an advanced precursor for CoA synthesis (**Figure 1C**). However, the ability of an organism (including Mtb) to obtain CoA through the salvage of PantSH depends on three factors: first, availability of PantSH in the environment; second, pathways by which it can be taken up; and third, ability of the organism’s PanK to act on PantSH as an alternative substrate to transform it directly into 4′-phosphopantetheine (P-PantSH, **7**), thereby bypassing the need for the reactions catalyzed by CoaBC (**Figure 1C**). In addition, it has been demonstrated that in some organisms P-PantSH can be taken up to allow for the formation of CoA only through the action of PPAT and DPCK (Srinivasan et al., 2015). Finally, Mtb’s DPCK is unique in that it is fused to a domain of unknown function, which could impact on its regulation and activity (Walia and Surolia, 2011). Clearly, the target assessment of individual CoA biosynthetic enzymes in Mtb is complex and cannot be reduced to a simple gene essentiality analysis.

### Hijacking CoA Biosynthesis: Using CoA Antimetabolites as Inhibitors

Apart from targeting one or more of the Pan or CoA biosynthesis enzymes directly for inhibitor development, a promising alternative is to use structural analogues of the respective pathway precursors, their intermediates or the final products, as inhibitors. Such an antimetabolite strategy is based on the closely related structures interfering with one or several CoA biosynthesis or CoA-dependent enzymes, leading to bacteriostasis or bactericidality (Shapiro, 2013). The effective use of antimetabolites as antibacterials dates back to Gerhard Domagk’s discovery of Protonsil (sulfanilamide) as the first synthetic antibacterial, as its structure resembles that of *p*-aminobenzoic acid (the natural substrate of DHPS) and therefore acts as a competitive inhibitor of the enzyme (Hammoudeh et al., 2013).

**Figure 2:**
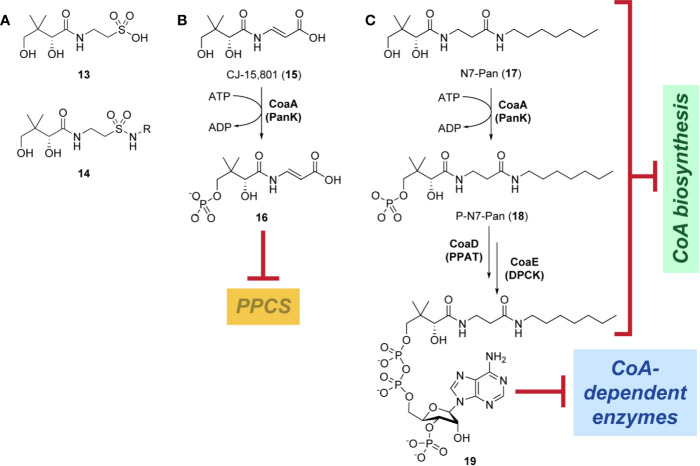
Pan and CoA antimetabolites. **(A)** Pantoyltaurine **13** and its amides **14** were some of the first Pan antimetabolites tested for antibacterial activity. **(B)** The fungal natural product CJ-15,801 **15** is a Pan antimetabolite that is activated via phosphorylation by PanK; its phosphate **16** subsequently inhibits PPCS. **(C)** Pan antimetabolites that are structurally similar to the CoA degradation product PantSH **6** can hijack the CoA salvage pathway enzymes to be transformed into CoA antimetabolites. For example, the pantothenamide N7-Pan **17** is phosphorylated by PanK to form **18**, which is then converted into **19** by PPAT and DPCK. These compounds can inhibit the biosynthesis of CoA in several ways, e.g., by competing with the natural substrates for access to the biosynthetic enzymes, by causing the build-up of biosynthetic intermediates that may regulate the enzymes through feedback inhibition, and by causing such feedback inhibition directly by mimicking the structures of the intermediate. Meanwhile, the CoA antimetabolites themselves can inhibit any CoA-dependent enzyme.

Indeed, following the discovery of the antifolates, several structural analogues of Pan were prepared with the view of achieving a similar outcome (Spry et al., 2008). This led to the discovery and testing of pantoyltaurine **13** and its amides **14** for antibacterial activity (**Figure 2A**). Interestingly, one of the results of this study was the discovery that many antimetabolites mimicking the structure of Pan are actually accepted and transformed by the CoA pathway enzymes to form active inhibitors (Moolman et al., 2014). In some cases, the target is one of the CoA biosynthetic enzymes. An example of this mode of inhibition is the fungal natural product CJ-15,801 **15** that inhibits PPCS after it has been phosphorylated by PanK to give **16** (**Figure 2B**). In other cases, the Pan antimetabolite is fully transformed by PanK, PPAT and DPCK into CoA antimetabolites that lack the crucial thiol group on which its activity is based. This is the situation for the *N*-heptyl pantothenamide N7-Pan **17** (**Figure 2C**). Consequently, such compounds can have a multi-pronged inhibitory effect: first, by competing with natural precursors of CoA during its biosynthesis, thereby reducing the rate of its formation, second by leading to the build-up of intermediates of the pathway that could allosterically inhibit the pathway enzymes through feedback mechanisms, third by acting as such feedback inhibitors themselves by mimicking the natural pathway intermediates, and lastly by forming CoA analogues that act as inhibitors of a range of CoA-dependent enzymes. Since CoA also serves as source of the 4′-phosphopantetheine group of the *holo*-acyl carrier protein (ACP) involved in fatty acid biosynthesis, CoA antimetabolites can have multifaceted impact on cellular physiology. However, the rational design of such inhibitors poses a special challenge as not only must their interactions with the putative CoA-utilizing targets be optimized, but the ability of their precursors to act as reasonable substrates for the CoA biosynthetic enzymes should also be considered. This difficult balancing act is not easily achieved.

### Providing a Context for CoA-Directed Antituberculosis Drug Development

As indicated above, while every enzyme taking part in Pan and CoA biosynthesis may present itself as a potential target for inhibitor development based on gene essentiality analysis, within the context of Mtb biology and the specific conditions under which Mtb survives and proliferates in the human host, not each enzyme is an equally vulnerable target. This review aims to not only give an overview of the inhibitor discovery work that has already been performed on each of the enzymes, but to also provide an assessment of their suitability as drug targets, *i.e.*, the likelihood that small molecule inhibitors could be translated into viable new antiTB therapies based on the insight gained from experimental studies performed on these proteins to date. Our target assessment leans heavily on studies of conditional knockdown mutants of the genes encoding each of the Pan and CoA biosynthetic enzymes as performed by Mizrahi and co-workers; these studies provide a more nuanced perspective on target vulnerability by studying the impact of reducing the endogenous levels of each of the enzymes in question on Mtb’s rate of growth and its virulence in mouse infection models (Abrahams et al., 2012; Evans et al., 2016). Our analysis also aims to complement that of a comprehensive *in silico* drug target identification pipeline in Mtb, called targetTB (Raman et al., 2008). This pipeline incorporates the analyses of the protein-protein interactome, the reactome’s flux balance, experimentally-derived phenotype essentiality data and the target sequences, as well as an algorithm-based structural assessment of targetability.

In addition, the potential of Pan and CoA antimetabolites as new antiTB drugs will also be highlighted, focusing on cases where CoA pathway enzymes have been shown to be inhibited by such compounds, or where they are known to be required for the metabolic activation of antimetabolite precursors to their active inhibitor forms. In light of the prevailing use of parameters reporting the inhibition of isolated enzymes (such as IC_50_ or *K*_i_) as the primary criterion or driver in inhibitor development and screening efforts, we also attempted to provide information on how well this translated to observed whole cell inhibition (as reported by minimum inhibitory concentrations, or MICs). As such, the overarching goal of this review is to provide a high-level view of antiTB drug development targeting Pan and CoA-related biology in Mtb.

## Targeting Pantothenate Biosynthesis

The discovery that a Pan auxotrophic Mtb strain is highly attenuated in immunocompromised mice led to the launch of several inhibitor discovery initiatives in the context of antiTB drug development. The majority of these were focused on the PanC enzyme, not only because the *panC* gene was one of those deleted in the Pan auxotroph, but also for medicinal chemistry reasons as detailed below. However, this section will describe the current status of our knowledge of each of the Mtb Pan biosynthetic enzymes as potential targets for drug development.

### PanB – Ketopantoate Hydroxymethyltransferase (KPHMT)

#### PanB Enzyme Structure and Mechanism

Ketopantoate hydroxymethyltransferase (KPHMT), also known as 3-methyl-2-oxobutanoate hydroxymethyltransferase in many databases, is encoded for by the *panB* gene (Rv2225) in Mtb to give the Mtb PanB enzyme (*Mt*PanB). It is the first enzyme in the Pan biosynthetic pathway and transforms α-ketoisovalerate (3-methyl-2-oxobutanoate, **8**) to ketopantoate (2-dehydropantoate, **9**) through transfer of a hydroxymethyl group provided by *N*_5_,*N*_10_-methylenetetrahydrofolate (*N*_5_,*N*_10_-CH_2_-THF) cofactor in the presence of a metal ion (Teller et al., 1976; Powers and Snell, 1976; Powers and Snell, 1979). The metal acts as a catalyst in the formation of the enol intermediate that leads to the formation of the new C–C bond. PanB has the greatest preference for Mg^2+^ but can also use other metals such as Mn^2+^, Zn^2+^, Co^2+^, Ni^2+^, and Ca^2+^ with the rate of enolization, *k*_enol_, decreasing by approximately a third for Zn^2+^ and Co^2+^ and nearly 15-fold for Ni^2+^ and 32-fold for Ca^2+^. The *k*_enol_ of α-ketoisovalerate was measured through monitoring the exchange of the β-hydrogen by NMR spectroscopy (Powers and Snell, 1976; Sugantino et al., 2003). From studies on the PanB enzymes from *Escherichia coli* and *Salmonella enterica* serovar Typhimurium, there is some evidence of feedback inhibition by end products within the pathway including Pan **2** (500 µM), CoA **1** (1 mM) and pantoate **5** (50 µM), as evidenced by increased *K*_M_ and lower V_max_ values at concentrations greater than those specified (Powers and Snell, 1976; Rubio and Downs, 2002). However, the physiological relevance of such a feedback inhibition mechanism requires further investigation and seems unlikely as these concentrations exceed those normally encountered in cells.

Three catalytic mechanisms have been proposed and are discussed and contrasted in a previous review of the pathway (Webb et al., 2004). The kinetic constants for *Mt*PanB have been determined by using a spectrophotometric assay that couples ketopantoate production to the consumption of NADPH by using the next pathway enzyme, ketopantoate reductase (PanE) (Sugantino et al., 2003). The determined parameters were a *K*_M_ (α-ketoisovalerate) of 240 µM and a *k*_cat_ value of 47 min^-1^, which is comparable to that seen for the *E. coli* enzyme (Powers and Snell, 1976; Sugantino et al., 2003).

**Figure 3:**
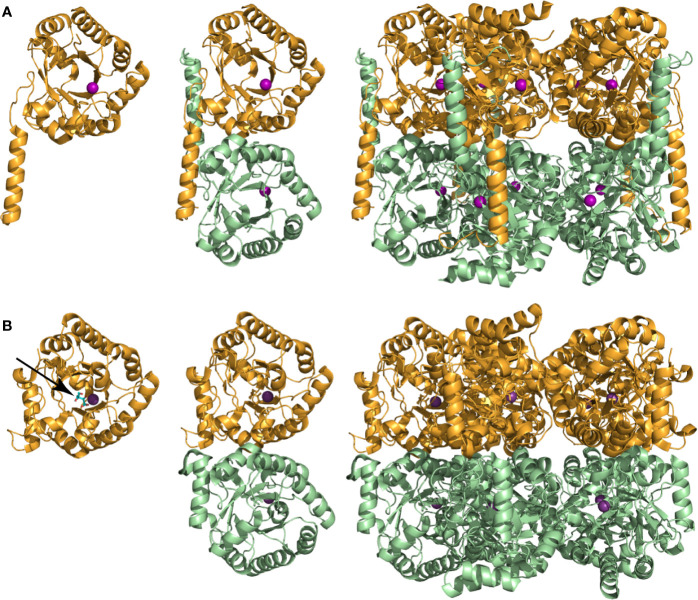
Subunit and oligomeric structures of PanB proteins. **(A)** Structure of *Mt*PanB (PDB: 1OY0) showing from left to right the monomer subunit, the interaction of two opposing subunits via the extended α-helix in the *C*-terminus, and the native decamer consisting of two stacked pentameric rings. A Mg^2+^ ion (magenta ball) bound at the top of the β/α (TIM) barrel defines the location of the active site. **(B)** Same views of *Ec*PanB (PDB: 1M3U) to highlight the differences in subunit interactions in the constitution of the native decamer. The stick structure of ketopantoate **9** bound in the active site of *Ec*PanB is indicated by an arrow.

Apart from the initial characterization studies, few other investigations on *Mt*PanB have been reported. Its crystal structure was solved in 2003 but since then no new data have been published (Chaudhuri et al., 2003). The enzyme’s quaternary structure exists as a decamer consisting of two pentameric rings stacked on top of each other (**Figure 3A**). Although based on the β/α (TIM) barrel fold, its structure contains several unique modifications (Sugantino et al., 2003; Chaudhuri et al., 2003). These include slightly different positioning of some of the α-helices that form the outer rim of the barrel and replacement of one helix by a coil. There are also modifications at the *N*-terminus. Additionally, one of the α-helices in the *C*-terminal region is slightly longer than usual and spans two different monomers from opposite pentameric rings. This domain swapping—which is unique among those PanB enzymes that have been characterized structurally—results in covering of the exposed hydrophobic surface of the opposing barrel, and is obvious when compared to the structure of the *E. coli* homologue, *Ec*PanB (**Figure 3B**). The active site lies at the top of the barrel within a deep cleft formed between the interface of the subunits from the pentameric ring and the residues here are highly conserved across the family (Chaudhuri et al., 2003).

#### Assessing PanB as a Drug Target

No known inhibitors of any PanB homologue have been identified or designed yet. The only compounds showing some inhibition of PanB’s activity are analogues of **8** that are missing either its carboxyl group, its α-carbonyl function or both methyl groups (Powers and Snell, 1976). The *in silico* target identification pipeline, targetTB, identified *Mt*PanB as a high confidence target (Raman et al., 2008); in support of this, knockdown of *panB* was shown to cause bacteriostasis (Evans et al., 2016). Taken together, this suggests potential for *Mt*PanB inhibitors as antiTB agents. However, any inhibitor design initiative would require careful elucidation of the active site binding interactions and the proposed feedback inhibition mechanisms. This is particularly important as the β/α (TIM) barrel fold is a common structural motif used by several enzymes that are mechanistically similar to PanB, suggesting that selective inhibition of *Mt*PanB may be difficult to achieve through strategies aimed only at the discovery of competitive inhibitors. Instead, compounds that exploit the proposed feedback mechanism could prove more valuable starting points, as these are more likely to be specific to *Mt*PanB.

### PanC – Pantothenate Synthetase (PS)

#### PanC Enzyme Structure and Mechanism

Pantothenate synthetase (PS), the product of the *panC* gene (Rv3602c), catalyzes the Mg^2+^- and ATP-dependent condensation of pantoate **5** and β-alanine **3** to form Pan **2** (**Figure 1A**) (Spry et al., 2008). Mechanistically, the PS-catalyzed reaction proceeds in two sequential steps. The first step involves activation of pantoate’s carboxylic acid through adenylylation. The resulting reactive pantoyl adenylate intermediate (**20**) subsequently undergoes nucleophilic attack by β-alanine’s amine in the second step, resulting in the release of AMP and the Pan product (Zheng and Blanchard, 2001; Pandey et al., 2018). Mtb PanC (*Mt*PanC) has been well characterized both structurally and kinetically, with crystal structures of the enzyme in its apo form, as well as in complex with several substrates, inhibitors, and reaction intermediates (Zheng and Blanchard, 2001; Wang and Eisenberg, 2003; Zheng et al., 2004; Wang and Eisenberg, 2006; Pandey et al., 2018). The enzyme’s activity is typically measured using an assay that couples the production of AMP to the oxidation of NADH using myokinase, pyruvate kinase and lactate dehydrogenase. Using this assay, Zheng and Blanchard confirmed that *Mt*PanC requires Mg^2+^ and has a Bi Uni Uni Bi Ping Pong kinetic mechanism similar to that reported for the *E. coli* homologue (Miyatake et al., 1978), with *K*_M_ (pantoate) of 130 µM, *K*_M_ (β-Ala) of 800 µM, *K*_M_ (ATP) of 2.6 mM and a *k*_cat_ value of 3.4 s^-1^ (Zheng and Blanchard, 2001).

The large amount of structural and mechanistic data available for *Mt*PanC has allowed elucidation of the active site and characterization of essential binding site interactions. Previous studies have confirmed that the enzyme is a member of the cytidylyltransferase superfamily (Wang and Eisenberg, 2003; Wang and Eisenberg, 2006). Its structure reveals a homodimer, with each subunit consisting of 290 amino acid residues totaling 33 kDa. The subunits have two defined domains, a large *N*-terminal domain that forms a Rossmann fold (residues 1–186), and a smaller *C*-terminal domain that incorporates a three-stranded antiparallel β-sheet with a helical layer above it (Wang and Eisenberg, 2003; Wang and Eisenberg, 2006).

As commonly seen in nucleotide binding proteins, the active site of *Mt*PanC is located on the *N*-terminal domain. It appears toward the end, within the central parallel β-sheet and is partially covered by β-strands from the *C*-terminal domain. The bottom of the active site is primarily hydrophobic, in contrast to the top half of the cavity, where several charged residues are located (Wang and Eisenberg, 2003). These residues are important for binding ATP, and also participate in catalysis. Additionally, four arginine residues covering the active site cavity form a positively charged region and are thought to attract the negatively charged substrates (i.e., ATP and pantoate) to the active site (Wang and Eisenberg, 2003).

Interestingly, in contrast to that seen for the *E. coli* homologue *Ec*PanC, the relative positions of the two domains of *Mt*PanC remain unchanged in structures of the enzyme in complex with eight different substrates/reaction intermediates. This difference was attributed to *Mt*PanC having extensive interactions between the *N*- and *C*-terminal domains, many of which would not be present in *Ec*PanC if it formed a similar closed conformation (Wang and Eisenberg, 2003). The consequence of this difference is that substrates can diffuse into the *Mt*PanC active site without the need of domain movement. In the case of *Ec*PanC, it was postulated that opening and closing of the active site occurs because of a hinged movement of its *C*-terminal domain. Conversely, it is possible that the flexible region of the *Mt*PanC *C*-terminal domain (residues 75 to 88) functions as a gate to its active site cavity (Wang and Eisenberg, 2003). This claim is supported by the observation that these gate residues are disordered when the *Mt*PanC active site is occupied by both AMPCPP and glycerol, or pantoate alone, but becomes ordered upon formation of the pantoyl adenylate intermediate (Wang and Eisenberg, 2003; Wang and Eisenberg, 2006).

#### PanC Inhibitors Developed Through Rational Design

**Figure 4:**
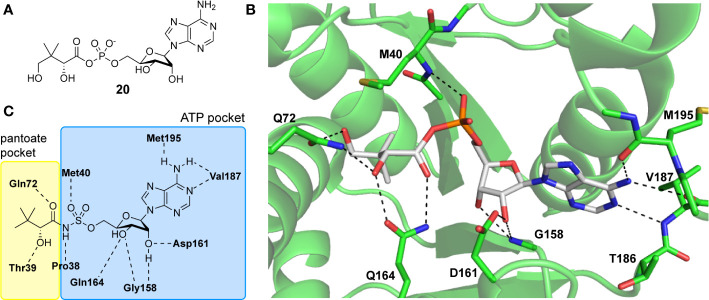
**(A)** Structure of pantoyl adenylate intermediate 20. **(B)** Binding interactions of pantoyl adenylate in the active site of *Mt*PanC [PDB: 1N2I (S. Wang and Eisenberg, 2003)]. **(C)** Hydrogen bonding interactions of compound 24 in the active site of *Mt*PanC, demonstrating the bisubstrate functionality of these compounds with the pantoate binding pocket shown in yellow and the ATP binding pocket shown in blue [figure modified from (Xu et al., 2014)].

The three-dimensional structure of *Mt*PanC in complex with pantoyl adenylate (**20**) demonstrates that the reaction intermediate has several polar interactions with active site residues (**Figure 4**). These strong interactions are essential in stabilizing the highly reactive intermediate, forcing the molecule to adopt a nearly linear conformation, thereby allowing it to adequately occupy the bottom of the active site cavity. This binding mode suggests that unreactive analogues mimicking the structure of the pantoyl adenylate reaction intermediate could act as potent inhibitors of *Mt*PanC with high affinity and specificity (Wang and Eisenberg, 2003; Ciulli et al., 2008; Xu et al., 2014).

There are two reported studies conducted to date that explore the use of unreactive pantoyl adenylate analogues as inhibitors of *Mt*PanC, one being a set of sulfamoyl adenylates, and the other a series of acyl-sulfamate and sulfamide mimetics (**Table 1**) (Ciulli et al., 2008; Xu et al., 2014). To prevent decomposition by intramolecular lactonization, all analogues lacked either the terminal hydroxyl or the carbonyl group of the pantoyl moiety. Co-crystal structures of *Mt*PanC complexed with each of three sulfamoyl adenylate analogues were reported by Ciulli et al. (2008). The authors found that the compound structurally most similar to the pantoyl adenylate intermediate (**20**) had the greatest potency across the two series, displaying nanomolar dissociation and inhibition constants. All three sulfamoyl analogues were found to act as competitive inhibitors towards pantoate. Likewise, the series of acyl-sulfamates and sulfamides reported by Xu et al. (2014) was also found to bind competitively with respect to pantoate.

**Table 1:** Structures and inhibition characteristics of *Mt*PanC inhibitors developed through rational design^a^.

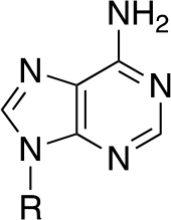			
Entry	R	*K*_i_ (µM)	MIC (µM)
**21**	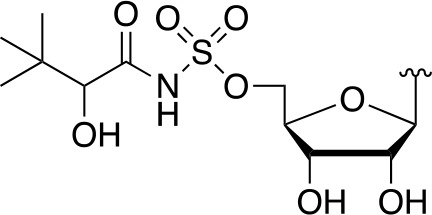	0.22 ± 0.03	–
**22**	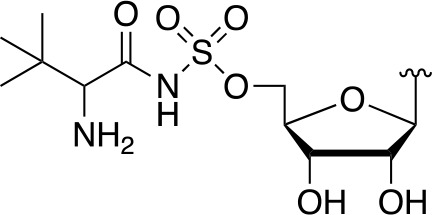	4 ± 0.6	–
**23**	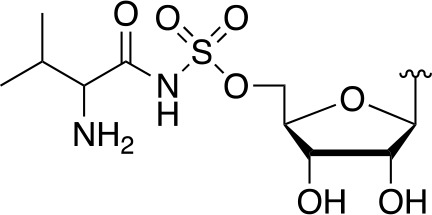	18 ± 3	–
**24**	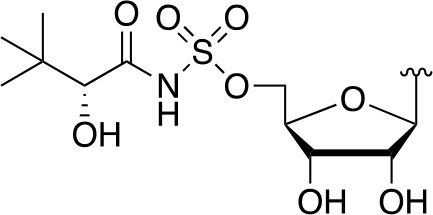	0.27 ± 0.04	> 250
**25**	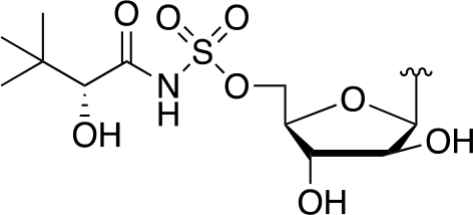	1.73 ± 0.24	> 250
**26**	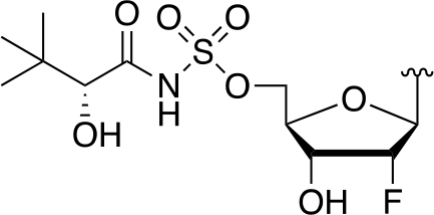	0.99 ± 0.30	> 250
**27**	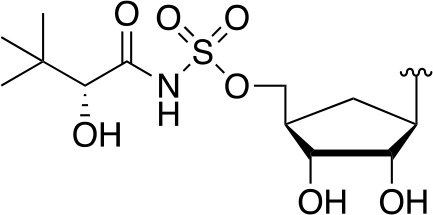	0.87 ± 0.12	> 250
**28**	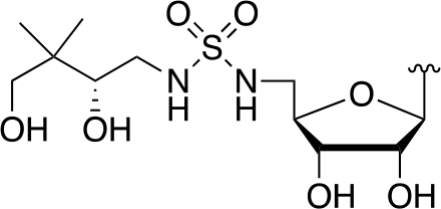	3.47 ± 0.48	> 250

^a^Compounds **21** – **23**, reported by (Ciulli et al., 2008), are competitive inhibitors with respect to ATP; MtPanC inhibition was determined using a PK/LD coupled kinase assay. Compounds **24** – **28**, reported by (Xu et al., 2014), are competitive inhibitors with respect to pantoate; MtPanC inhibition was determined using the MesG coupled assay and Mtb whole-cell screening was carried out against wildtype H37RvMA and a MtPanC-depleted strain. All values are shown as reported in the original studies.

The co-crystal structures produced by Ciulli et al. (2008) allowed for binding mode elucidation and identification of many key binding interactions within the pantoate pocket. The carbonyl and hydroxyl groups of compound **21** (**Table 1**) form hydrogen bonds with two conserved glutamine residues at the pantoate binding site. These interactions are not observed with related derivatives in this series (compounds **22** and **23**), resulting in a 10-fold reduction in binding affinity. The authors postulate that this can be attributed to the exchange of the hydroxyl group with an amine in compounds **22** and **23**, as the introduction of the amine group changes the hydrogen bond donor/acceptor interactions with the two glutamine residues (Ciulli et al., 2008). Consequently, this leads to a disruption of the hydrogen bond network between three active site residues that are conserved across all bacterial PS enzymes and are known to be essential for enzyme activity (Zheng et al., 2004). Through further structural elucidation, a number of other key features within the pantoate pocket were identified, with plans to test the most potent compound for activity in a cell-based assay against Mtb (Ciulli et al., 2008).

In comparison, the series of adenylate mimics synthesized by Xu et al. (2014) were designed to function as bisubstrate inhibitors that bind both the pantoate and ATP binding pockets (compounds **24-28**, **Table 1**). These inhibitors were designed to use an acyl-sulfamate or -sulfamide as a bioisosteric replacement for the labile acyl phosphate moiety present in the native intermediate. In addition, the inhibitors display structural modifications at the terminal hydroxyl group, carbonyl group, and ribose ring. Compound **24**, with an acyl-sulfamate linkage and a natural ribose moiety, was found to be the most potent, while sulfamide **28** was the least potent. Modifying the ribose moiety in **24** to arabinose (compound **25**) led to a marked loss in affinity. Molecular docking studies revealed that high binding affinity is contingent upon the presence of the secondary hydroxyl and carbonyl groups, which are necessary in forming critical hydrogen bond contacts with the two highly conserved glutamine residues at the pantoate binding site, a finding in agreement with previous studies (Ciulli et al., 2008; Xu et al., 2014). Unfortunately, the compounds in this study were inactive against Mtb in whole cell assays, as well as against a *panC* knockdown strain (Xu et al., 2014). The authors attribute this lack of potency to poor cell penetration or possible efflux as the most probable causes, concluding that PanC may not be an ideal antimycobacterial drug target due to low vulnerability to inhibition.

A fragment-based approach that combines both fragment growing and fragment linking methods in the development of *Mt*PanC inhibitors has been employed by a number of groups (Hung et al., 2009; Sledz et al., 2010; Silvestre et al., 2013). For example, the fragment growing approach was used by Hung et al. (2009) who elected to initiate the process using a methoxy-substituted indole derivative (compound **29**), known to act as an ATP-competitive ligand (**Table 2**). Incremental structural modifications were made to compound **29** at the C2 and N1 positions of the indole ring, and the resulting compounds were evaluated in relation to the initial fragment. After several constructive iterations, this method led to the identification of the acyl sulfonamide inhibitor **30** with a *K*_i_ value of 27 µM. A fragment linking approach was used when it was found that benzofuran analogue **31**, which was first identified from a thermal-shift screen, binds to the active site of *Mt*PanC in a considerably different manner than compound **29**. The ternary complex structure of *Mt*PanC and both compounds **29** and **31** showed little movement of the molecules in relation to their initial positions, and minor conformational changes of the target, suggesting that they may be linked to obtain a higher affinity ligand. To join the two fragments—while maintaining their initial binding modes—a variety of linkers were introduced and assessed. A significant increase in potency was observed when an acyl sulfonamide linker was used, giving rise to analogue **32** that had a *K*_i_ value of 9 µM and a ligand efficiency (LE) of 0.26. Interestingly, the indole ring and the acyl sulfonamide present in both compounds **30** and **32** were found to bind in an identical manner, whereas the 4-methylpyridine ring in compound **30** occupies the β-alanine binding pocket, and the benzofuran ring in compound **32** binds to the pantoate pocket. Overall, the discovery of the two remarkably similar compounds with comparable potencies demonstrated that the two different fragment-based ligand discovery strategies—fragment-growing and fragment-linking—can successfully be applied to the development of inhibitors of *Mt*PanC (Hung et al., 2009). Unfortunately, none of these compounds showed inhibition of the growth of wild-type Mtb, but all inhibited the *panC* conditional mutant, confirming that they were on target. Compound **32** showed the strongest effect, correlating with its activity as the most potent *Mt*PanC inhibitor (Abrahams et al., 2012).

**Table 2:** Structures and inhibition characteristics of *Mt*PanC inhibitors developed through fragment-based approaches^a^.

Entry	Structure	LE	*K*_D_ (µM)	Identification
**29**	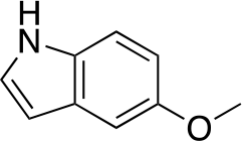	0.37	1100	WaterLOSY NMR screening
**30**	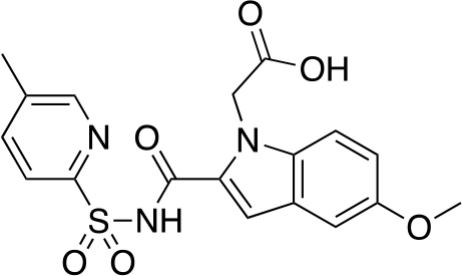	0.28	1.5	Fragment growing
**31**	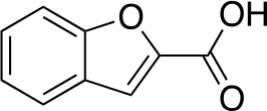	0.34	1000	Thermal-shift screen
**32**	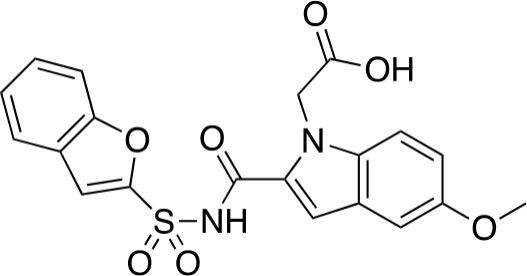	0.26	1.8	Fragment linking
**33**	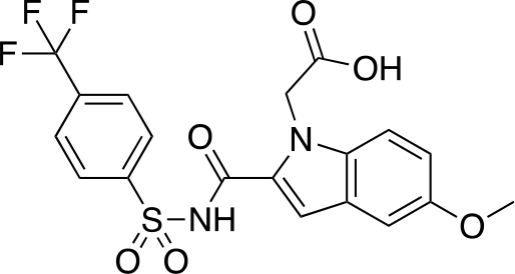	0.30	0.2	Group efficiency analysis
**34**	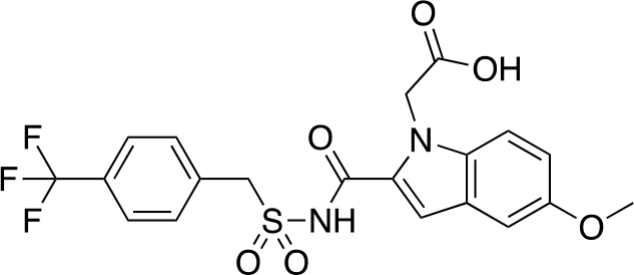	0.28	–	Group efficiency analysis

^a^Compounds **29**–**32** were reported by (Hung et al., 2009) and **33** and **34** were reported by (Hung et al., 2016). Both series of compounds are competitive inhibitors with respect to ATP, and K_D_ values were determined from titration experiments using ITC.

In a second paper, Hung et al. (2016) further optimized compounds **30** and **32** by implementing a group efficiency (GE) analysis, allowing different structural components within each molecule to be more closely examined and the binding contributions (ΔΔG) to be determined. As a result, this approach was used to efficiently highlight specific moieties within the molecules responsible for inefficient binding, enabling further modifications. Both the indole fragment and acetate substituent were shown to contribute significantly to overall binding. In contrast, insufficient binding contributions were identified for the methyl pyridine and benzofuran groups, thus replacement of these moieties was explored. The acyl sulfonamide linker also showed limited contributions to binding; however, this group is essential to retain the non-hydrolyzable linkage and was thus retained. A series of ten indole acyl sulfonamides was synthesized and evaluated, resulting in several compounds with sub-micromolar activity against *Mt*PanC. In particular, compound **33** (**Table 2**), with a highly electronegative trifluoromethyl substituent, was found to be the most potent inhibitor in this series with a *K*_D_ value of 200 nM and an IC_50_ value of 5.7 µM. Additional crystallographic studies of the four most potent compounds were used to further probe the deep pocket of the *Mt*PanC P1 site. The authors hypothesized that the addition of a methylene group between the sulfonyl and aromatic moieties would facilitate the movement of the aromatic ring to below Met40, causing a substituent in the *para* position to be pushed into the back of the P1 pocket (Hung et al., 2016). This led to the design of compound **34** (**Table 2**), a *para-*trifluoromethyl-substituted benzylsulfonamide, which showed favorable hydrophobic bonding interactions with residues deep in the P1 site, confirming the anticipated inhibitor requirements for this favorable binding orientation to occur. Compound **34** showed a significant increase in potency against *Mt*PanC in comparison to **33**, with an IC_50_ value of 250 nM. This study demonstrates the utility of a fragment-based approach proceeded by lead optimization of hit fragments in developing potent inhibitors. Furthermore, the ability to identify the binding distribution of a given compound based on GE analysis allows for the modification of specific groups, thus leading to more potent inhibitors.

In a more recent structure-based study, a series of thiazolidine derivatives as inhibitors of *Mt*PanC were developed using energy-based pharmacophore (e-pharmacophore) modeling (**Table 3**). Devi et al. (2014) began by using the crystal structure of *Mt*PanC in complex with pantoyl adenylate to generate an energetically optimized, structure-based pharmacophore by using Phase software (Dixon et al., 2006). This e-pharmacophore was then used to carry out a high throughput virtual screen of a 500,000-compound library. Selected hits were synthesized and tested *in vitro* against *Mt*PanC, where seven compounds displayed > 60% inhibition at 25 µM. The most potent was compound **35**, a thiazolidine derivative containing a benzodioxo group and a 3-pyridyl moiety, with an IC_50_ of 1.12 µM (Devi et al., 2014). Further analysis revealed four hydrogen-bonding interactions and polar contacts between **35** and active site residues of *Mt*PanC, as well as a similar overall orientation to that of the native ligand. A second set of 19 compounds were designed and synthesized in order to explore the SAR around compound **35**, in which the benzodioxo position was modified with a variety of substituted phenyl rings, and the 3-pyridyl group replaced with phenyl, naphthyl, and other pyridyl groups. Many of the compounds displayed submicromolar activity against *Mt*PanC, while only six compounds had MIC values <10 µM (Devi et al., 2014). Two of the most active inhibitors (compounds **36** and **37**) were selected to undergo further investigation. These compounds were found to display improved activity against *Mt*PanC (IC_50_ value of 350 nM for **36**) and against Mtb cells (MIC value of 2 µM for **36**). In addition and importantly, compounds **36** and **37** also display activity against dormant Mtb cells (Devi et al., 2014).

**Table 3:** Structures and inhibition characteristics of *Mt*PanC inhibitors developed through energy-based pharmacophore modeling^a^.

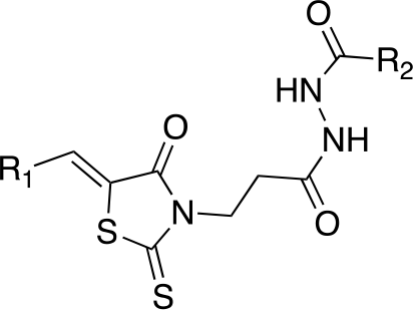				
Entry	R_1_	R_2_	IC_50_ (µM)	MIC (µM)
**35**	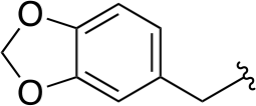	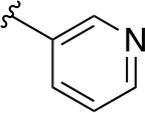	1.12 ± 0.12	54.81
**36**	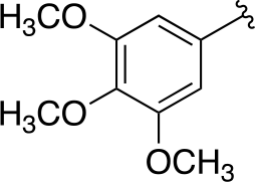	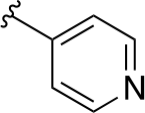	0.35 ± 0.01	1.55
**37**	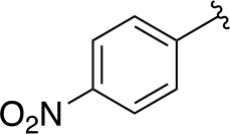	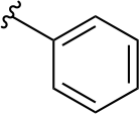	0.37 ± 0.02	1.71

^a^Compounds **35–37** were reported by (Devi et al., 2014). MtPanC inhibition was determined using a PK/LD coupled kinase assay, and Mtb whole-cell screening was carried out against wildtype H37Rv cells by using the MABA method. All values are shown as reported in the original study.

#### PanC Inhibitors Identified by Screening

Several inhibitors that target *Mt*PanC have been identified through various screening methods (White et al., 2007; Velaparthi et al., 2008; Yang et al., 2011; Kumar et al., 2013; Ntie-Kang et al., 2013; Samala et al., 2013; Naidu et al., 2014; Samala et al., 2014; Naidu et al., 2016; Naidu et al., 2016; Amaroju et al., 2017; Pradhan and Sinha, 2018; Kumar et al., 2019; Hassan et al., 2020; Singireddi et al., 2020). White et al. (2007) developed an automated high-throughput screen (HTS) by adapting the coupled assay first used by Zheng and Blanchard (2001), and used it to identify known drugs that inhibit *Mt*PanC. A diverse library of 2880 compounds containing drugs across a wide range of therapeutic areas, experimentally bioactive molecules, and natural products was surveyed. Twenty-nine compounds showed some activity based on IC_50_ values; these were rescreened against the coupling enzymes to confirm their specificity for *Mt*PanC. Only nafronyl oxalate (compound **38**, **Table 4**) was identified as a specific inhibitor of *Mt*PanC, showing competitive inhibition with a *K*_i_ of 75 µM, one-fifth that of the substrate’s *K*_M_. A co-crystal structure of *Mt*PanC with **38** was compared to the crystal structure of *Mt*PanC complexed with pantoyl adenylate. This showed that the two molecules were positioned similarly in the active site of *Mt*PanC, but a more favorable hydrogen-bonding network was established between the pantoyl adenylate intermediate and the *Mt*PanC active site. Unfortunately, even though this compound showed promising competitive inhibition, it was found to be inactive against Mtb in a whole cell assay.

**Table 4:** Structures and inhibition characteristics of *Mt*PanC inhibitors developed through high throughput screening^a^.

Entry	Structure	*K*_i_ (µM)	IC_50_ (µM)	MIC (µM)
**38**	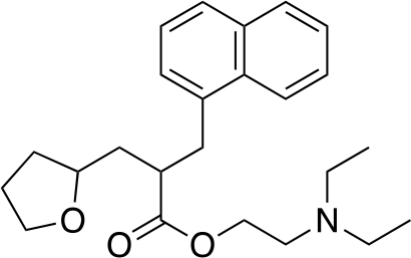	75 ± 13	–	> 13
**39**	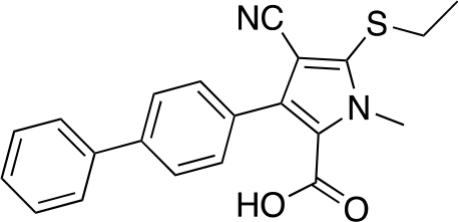	1.8 ± 1.1^b^	0.174 ± 0.02	115
**40**	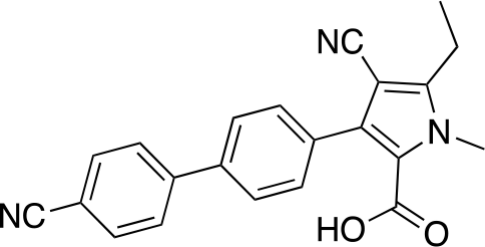	4.0 ± 1.1^b^	0.297 ± 0.037	54
**41**	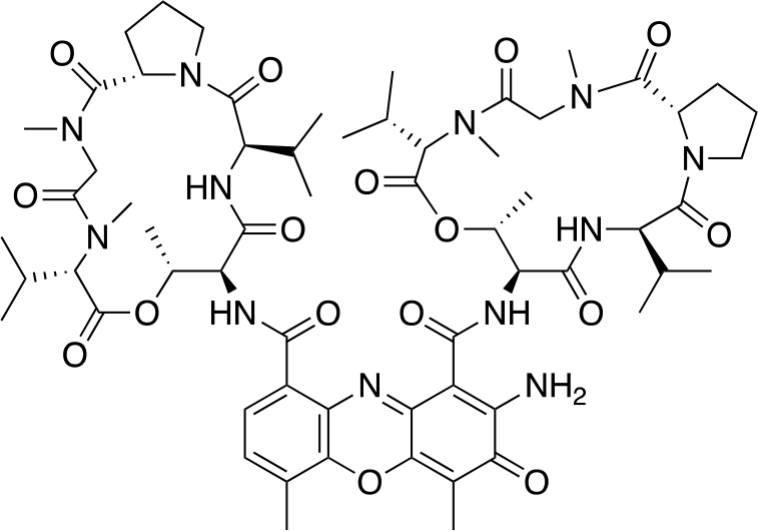 (actinomycin D)	–	250.72 ± 39.69	–
**42**	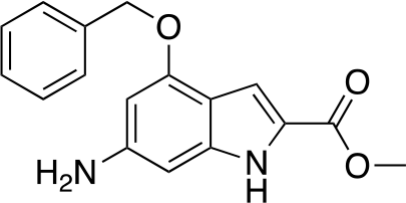	1.4	22.44 ± 1.55	54

^a^Compound **38**, reported by (White et al., 2007), is a competitive inhibitor with respect to ATP; MtPanC inhibition was determined using a PK/LD coupled kinase assay. Compounds **39** and **40**, reported by (Kumar et al., 2013), are competitive inhibitors with respect to ATP as determined using the same coupled assay. Mtb whole-cell screening was carried out against wildtype H37Rv cells and against a MtPanC conditional knockdown strain. Compounds **41** and **42** are reported by (Yang et al., 2011). Compound **42** is a competitive inhibitor with respect to ATP and Mtb whole-cell screening was carried out against wildtype H37Rv cells. All values are shown as reported in the original studies.

^b^EC_50_ values are reported.

In a similar set of studies, Kumar et al. (2013) developed an enzyme-based assay that modified previously established PanC coupled assays relying on absorbance. The standard coupled assay was modified to account for any autofluorescence occurring in compound libraries by generating a fluorescent signal that could be measured as the endpoint of the assay. This assay was then used to carry out an HTS on a large and extensive library of nearly 100K compounds. Using this screen, two 3-biphenyl-4-cyanopyrrole-2-carboxylic acid analogues were selected for further evaluation (compounds **39** and **40**, **Table 4**). Both compounds were active against purified recombinant *Mt*PanC and were shown to inhibit growth of Mtb, however both also displayed some degree of toxicity. Conditional knockdown studies were conducted to determine the specificity of compounds **39** and **40**. The results were consistent with PanC-mediated inhibition, attributing the growth inhibition of Mtb to on-target activity against PanC. This data suggested that this scaffold could be further developed for improved potency against Mtb and decreased toxicity to mammalian cells (Kumar et al., 2013).

More recently, a molecular docking approach was employed by Pradhan and Sinha (2018) to screen a library of 154 small molecule containing amides known to inhibit pantothenate synthetase. *In silico* predictive results from this screen identified a sulfonamide analogue theorized to inhibit the enzyme, exhibiting significantly better docking scores than two approved sulfa drugs. Unfortunately, due to the lack of *in vitro* results, this compound could not be analyzed in relation to other similar compounds. A pharmacophore model was generated based on enzyme-ligand interactions established in the docking studies, providing insight into the binding mode of the sulfonamide analogue in the active site of *Mt*PanC. Furthermore, a molecular dynamics simulation study was carried out, with the corresponding predicted energy profile signifying the formation of a stable conformation upon enzyme-ligand binding (Pradhan and Sinha, 2018). In conjunction, the use of computational predictions as well as the continued exploration of amide analogues has further contributed in the development of novel antituberculars (Pradhan and Sinha, 2018).

In another virtual screening study, Yang et al. (2011) developed a novel HTS approach to discover potent *Mt*PanC inhibitors. They began by screening a small (~3000) library of compounds in a manner similar to the HTS performed by White et al. (2007), and identified actinomycin D (compound **41**, **Table 4**) as a weak inhibitor of *Mt*PanC, with an IC_50_ of 251 µM (Yang et al., 2011). To discover more potent inhibitors, the molecular mechanism of inhibition of **41** was determined by circular dichroism and fluorescence quenching. This allowed for the construction of a pharmacophore, followed by a virtual screening of 20K compounds based on these identified structural features. This approach led to the identification of a potent indole-containing *Mt*PanC inhibitor (compound **42**, **Table 4**) with an IC_50_ of 22 µM, a ten-fold improvement in activity compared with compound **41** (Yang et al., 2011). Additionally, compound **42** was active against wild-type Mtb, with an MIC of 54 µM. Collectively, this study details a new method, based on confirmed inhibitor-enzyme interactions, for exploring potential lead compounds.

As many current antitubercular drugs contain some form of nitrogen heterocycle, a series of hybrid molecules containing a pyrazine scaffold was recently investigated by Hassan et al. (2020). The compounds were designed to incorporate a pyrazine ring with either a carbohydrazide linker or a bioactive heterocyclic moiety. A preliminary *in silico* screen solely based on the structural formula of these compounds was performed to predict their biological potential. The predicted hits were tested *in vitro* against Mtb H37Rv using the microplate alamar blue assay (MABA), resulting in the identification of six compounds that displayed significant activity, with MIC values ranging from 0.78 µg/mL to 6.25 µg/mL and no cytotoxicity (compounds **43**–**48**, **Table 5**) (Hassan et al., 2020). Further investigation of these structures revealed that the compounds containing a carbohydrazide linker between the pyrazine ring and a second heterocyclic moiety exhibited the greatest potency against Mtb. A target fishing approach was employed on the most active analogue (compound **44**) to elucidate the mechanism of action and identify the target responsible for the observed whole cell activity. This was carried out computationally using a shape-based similarity study, pharmacophore mapping, and inverse docking. Together, the results of these studies suggest that *Mt*PanC is the target responsible for the activity of these inhibitors (Hassan et al., 2020).

**Table 5:** Structures and inhibition characteristics of *Mt*PanC pyrazine analogues developed through *in vitro* screening^a^.

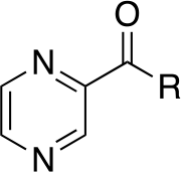		
Entry	R	MIC (µM)
**43**	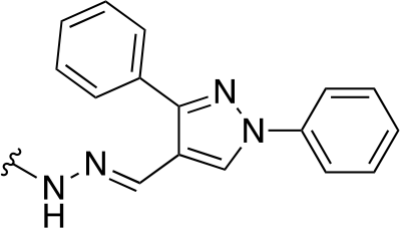	8.47
**44**	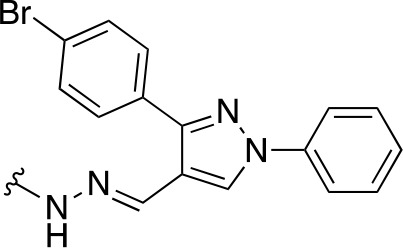	1.74
**45**	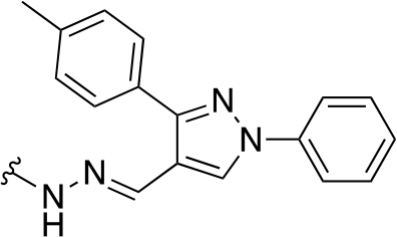	4.08
**46**	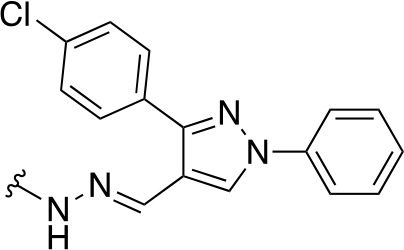	3.48
**47**	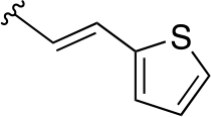	28.90
**48**	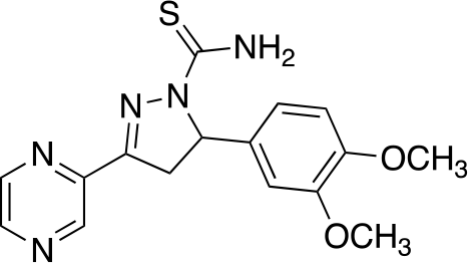	9.09
**49**	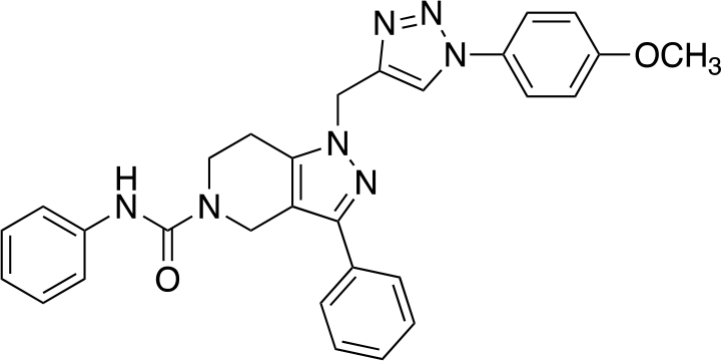	24.72

^a^Compounds **43–48**, reported by (Hassan et al., 2020), were evaluated for in vitro activity against Mtb H37Rv strain using the MABA method. Compound **49** was reported by (Amaroju et al., 2017) and evaluated against Mtb H37Rv strain (as well as two strains extracted from tuberculosis patients, the results of which are not shown here). All values are shown as reported in the original studies.

In a similar study, Amaroju et al. (2017) developed a series of compounds by implementing a molecular hybridization approach based on the structures of two previously reported *Mt*PanC inhibitors (Samala et al., 2014; Naidu et al., 2016). In prior work, twenty-six pyrazole and triazole compounds were synthesized. Compounds were tested for *in vitro* enzyme inhibition against *Mt*PanC and *in vitro* whole cell activity against three Mtb strains. The most active compound contained a 4-methoxyphenyl substituent on the triazole moiety (compound **49**, **Table 5**) with an IC_50_ of 1.01 µM and an MIC of 25 µM against wild-type Mtb (Amaroju et al., 2017). Consequently, compound **49** was found to be more active against both *Mt*PanC and Mtb whole cells than the pyrazole derivative after which it was designed. This potency, in combination with the low cytotoxicity displayed, shows the potential for further antimycobacterial development based on this core structure (Amaroju et al., 2017). The triazole scaffold was further explored more recently, where a series of molecules linking a quinazolinone to a 1,2,3-triazole were examined (Kumar et al., 2019). Unfortunately, these compounds displayed only moderate activity against Mtb.

#### Assessing PanC as a Drug Target

In summary, although an extensive amount of structural and mechanistic information is available for *Mt*PanC, no adequate clinical candidates have been reported to date. Many of the compounds designed and evaluated have shown low to submicromolar inhibition of *Mt*PanC, but overall failed to establish significant whole cell inhibition. This outcome may be explained by the results of the targeted knockdown studies of the Pan and CoA biosynthetic enzymes, which showed that reducing *Mt*PanC levels to >95% slowed cell growth, but did not arrest it (Abrahams et al., 2012). This would indicate that *Mt*PanC is a relatively invulnerable target in the context of Mtb drug development, and that *Mt*PanC-directed inhibitors would have to show near complete inhibition of the enzyme’s activity before it would impact on cell growth. Nonetheless, the compounds discussed in the studies above have provided a considerable foundation for development of inhibitors active against Mtb. Moreover, as the targetTB comprehensive *in silico* target identification pipeline recognized *Mt*PanC as a high confidence target, interest in this enzyme remains high (Raman et al., 2008). Within this context *Mt*PanC remains a viable and attractive target for selective antitubercular drug development, with the important caveat that prospective inhibitors would have to show high affinity binding or long residence times before they could be considered for further investigation.

### PanD – Aspartate Decarboxylase (ADC)

#### PanD Enzyme Structure and Mechanism

PanD, or aspartate decarboxylase (ADC), catalyzes the formation of β-alanine **3** from l-aspartate **4** *via* a decarboxylation reaction as its name implies (**Figure 1A**). Unlike most other decarboxylases that depend on a pyridoxal phosphate (PLP) cofactor for catalysis, PanD is one of a few enzymes that make use of an enzyme-bound pyruvoyl-group that is revealed after a post-translational cleavage step. In the case of Mtb PanD (*Mt*PanD, encoded by *panD* (Rv3601c)), the protein is first expressed as a 15.95 kDa pro-enzyme known as the π-protein that undergoes autocatalytic cleavage to form the active enzyme consisting of a smaller β-subunit and a larger α-subunit with the *N*-terminal pyruvoyl group (Chopra et al., 2002; Gopalan et al., 2006). The π-protein consists of seven β strands, two 3_10_ helices and two α helices that form a double ψ, β barrel (Gopalan et al., 2006). The autocatalytic cleavage of the π-protein occurs optimally at 37°C and goes to completion after 48 hours. As this roughly mirrors the doubling time and growth temperature of Mtb, the maturation process has been suggested as a mechanism of regulating the production of β-Ala **3** (Chopra et al., 2002). The crystal structure of the unprocessed π-protein is available (PDB ID: 2C45), and the mechanism of cleavage, which occurs at an internal serine, has been elucidated (Ramjee et al., 1997; Albert et al., 1998; Gopalan et al., 2006; De Villiers et al., 2010).

**Figure 5:**
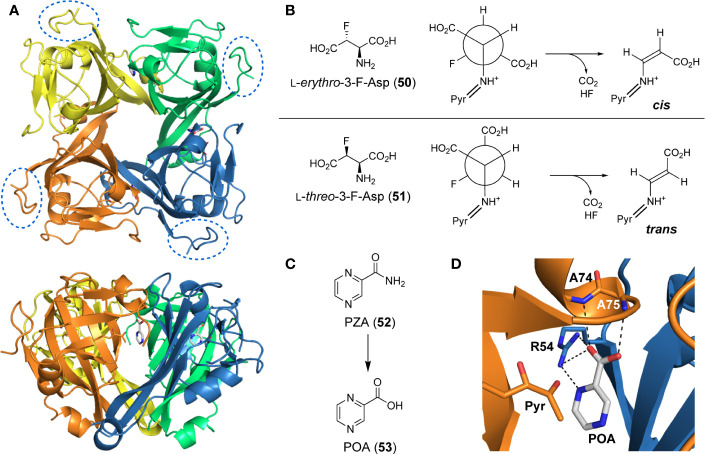
PanD structure and inhibitors. **(A)** Top and side views of the tetrameric structure of *Mt*PanD as determined by X-ray crystallography (PDB: 6OYY), with each processed π-protein (*i.e.*, consisting of α- and β-subunits) shown in a different color. The *C*-terminal extension, which is unique to *Mt*PanD among PanD proteins, is not resolved in the determined structure, but would be attached to the portion indicated in the dashed oval. **(B)** 3-Fluorinated l-aspartates (**50** and **51**) act as alternative substrates of *Mt*PanD, but undergo only single turnover followed by fluoride elimination. Depending on the epimer used, this results in the formation of either *cis-* or *trans*-enamines, which are transiently trapped in the enzyme’s active site. **(C)** Structure of the antiTB agent pyrazinamide (PZA, **52**), which is activated through hydrolysis to form pyrazinoic acid (POA, **53**). **(D)** Active site of *Mt*PanD showing the binding mode of POA (PDB: 6OYY); its hydrogen bonding interactions with the indicated active site residues are shown with black dashed lines. The pyruvoyl group (Pyr) that is required for catalysis is shown in the left foreground.

Interestingly, while size-exclusion chromatography (SEC) and X-ray crystallographic analysis indicate that *Mt*PanD exists as a tetramer (**Figure 5A**) (Gopalan et al., 2006; Sun et al., 2020), small angle X-ray scattering (SAXS) studies show that it forms a monodispersed octamer (stacked tetramers) in solution (Gopal et al., 2020). The Arg54 residue along with the pyruvoyl group are key actors within the active site, which is found at the interface of adjacent subunits of the tetramer. Specifically, Arg54 aids in substrate recognition through formation of a salt bridge with Asp’s β-carboxylate, while the pyruvoyl group forms a transient imine with its amino group to serve as an electron sink that facilitates its decarboxylation (Sharma et al., 2012a; Sharma et al., 2012b).

*Mt*PanD’s steady state kinetic parameters were determined by utilizing a discontinuous assay that monitors the conversion of **4** to **3** through generation of fluorescamine derivatives of each species, followed by HPLC analysis (Ramjee et al., 1997; Chopra et al., 2002). The *K*_M_ was found to be 219.6 µM and the *k*_cat_ 0.65 s^-1^.

#### PanD Inhibitors Developed Through Rational Design

De Villiers et al. (2010) set out to design mechanism-based inhibitors that capitalize on the differences between PLP- and pyruvoyl-dependent decarboxylases to obtain compounds that are selective for *Mt*PanD. For this purpose, they tested l-*erythro*-3-fluoro-asparate (**50**) and its l-*threo* epimer (**51**) and found that instead of being turned over catalytically, these substrate analogues underwent single-turnover decarboxylation, followed by elimination of fluoride (**Figure 5B**). This sequence produced enamine adducts that could be trapped within the active site (De Villiers et al., 2010). When the l-*erythro* epimer **50** was used, the resulting adduct was stable enough that it could be observed even without trapping; the result was rationalized based on the presence of the fluorine causing stabilization of the enamine adduct. However, in a kinetic assay, only the l-*threo* epimer **51** showed inhibition of *Mt*PanD activity because only its conformation was predicted to closely mimic that of the natural substrate when bound to the active site. Unfortunately, insufficient material was produced to test whether these compounds showed any activity against Mtb in growth assays; however, considering the reactive nature of α-fluorinated amino acids, it is likely that this compound would also affect other targets.

#### PanD Inhibitors Identified by Screening

Sharma and co-workers used a cheminformatics approach to identify potential inhibitors of *Mt*PanD (Sharma et al., 2012b; Sharma et al., 2012a). Using crystal structures of PanD enzymes from *Thermus thermophilus* and *Helicobacter pylori* with substrates bound in the active site, they found that these overlapped with the active site of the processed *Mt*PanD to within 1 Å. They subsequently used the Schrödinger software suite to perform a high-throughput virtual screen of compounds for binding to the model of the *Mt*PanD active site. Three public ligand libraries (Maybridge, NCI and FDA) totaling over 333K compounds were used in the screen, which resulted in the identification of 803 hits. A more refined screen was performed using Glide’s Extra Precision (XP) mode, resulting in 28 compounds being selected for pharmacokinetics analysis using Qikprop. From these, seven lead compounds were identified as the most suitable candidates for further testing and investigation (Sharma et al., 2012b; Sharma et al., 2012a). An additional seven inhibitors previously identified against *Ec*PanD were also tested (Williamson and Brown, 1979; Sharma et al., 2012a). While prior inhibition assays made use of the previously mentioned discontinuous assay that depends on fluorescamine derivatization of reaction mixtures, a simpler, direct and label-free assay was used for this study that relied on an NMR spectroscopy-based protocol that measured the formation of β-Ala **3** from l-Asp **4** directly (Sharma et al., 2012a). All inhibitors were tested at 1 mM using 1 mM l-Asp and 2.83 μM *Mt*PanD, and results were processed by comparing the % conversion of the inhibited reaction at the time point at which the uninhibited reaction reached 50% conversion. *K*_rel_ was subsequently calculated by comparing the ratio of % conversion with and without inhibitor (if unchanged *K*_rel_ = 1.0). The six most promising inhibitors identified in this manner (those with *K*_rel_ ≤ 0.4) were all simple organic acids, including oxaloacetate (the most potent of the inhibitors tested), β-hydroxyaspartate, l-glutamate and both d- and l-tartrate. The observed inhibition was rationalized to be mainly due to competitive binding to the enzyme active site through formation of non-specific electrostatic interactions.

#### PanD as a Target for Pyrazinamide

Pyrazinamide (PZA, **52**) has long been used as part of the standard combination therapy to treat Mtb. However, its primary target has yet to be definitively confirmed. A detailed review describing the current knowledge of PZA’s mode of action (MoA) (Gopal et al., 2019) and a paper that analyses the various proposed mechanisms and results (Anthony et al., 2018) have recently been published. Here, the focus will be on the information pertinent to *Mt*PanD, considering several recent findings that have highlighted its involvement in PZA’s MoA.

PZA is a prodrug and is activated by the pyrazinamidase PncA to pyrazinoic acid (POA, **53**) (**Figure 5C**) (Scorpio and Zhang, 1996; Via et al., 2015; Lanoix et al., 2016; Yadon et al., 2017; Naftalin et al., 2017). A variety of targets have been suggested for PZA/POA throughout the years; however, there is an increasing amount of evidence that *Mt*PanD is likely at least one of these targets. This is supported by both the *in vitro* and *in vivo* isolation of resistant mutants of PZA with missense mutations in *panD* (Shi et al., 2014; Gopal et al., 2016; Gopal et al., 2017b; Gopal et al., 2019). Two main proposals have been made on how the active inhibitor (POA) affects *Mt*PanD activity (Gopal et al., 2019). The first suggests that POA binding to *Mt*PanD inhibits its ADC activity, leading to an observed reduction in the production of β-Ala, and consequently in Pan and CoA levels (Gopal et al., 2016; Gopal et al., 2017a). This proposal is supported by several studies confirming the binding of POA to *Mt*PanD (Shi et al., 2014; Dillon et al., 2014; Gopal et al., 2017a), including a crystal structure showing POA bound in the enzyme’s active site (**Figure 5D**) (Sun et al., 2020). This proposed MoA is supported by the finding that the two best-studied resistant mutations cause amino acid exchanges in the enzyme’s active site loops (Zhang et al., 2013; Shi et al., 2014; Gopal et al., 2017b). The second proposal is based on the finding that PZA resistant mutants also showed missense mutations in the gene encoding the unfoldase ClpC1, suggesting that upon binding of POA, *Mt*PanD’s *C*-terminal protease degradation tag is exposed to a greater extent (**Figure 5A**) (Gopal et al., 2019; Gopal et al., 2020). Since ClpC1 binds and transports proteins to the ClpP protease, increased exposure of the tag leads to a greater amount of *Mt*PanD to be recognized by ClpC1 and degraded by ClpP (Raju et al., 2012; Yamada and Dick, 2017; Gopal et al., 2019; Gopal et al., 2020).

Whether PanD is the primary target of PZA (**52**) remains a matter of debate for several reasons. First, the incidence of PZA resistant clinical isolates with mutations in *panD* is low (Maslov et al., 2015; Werngren et al., 2017; Coll et al., 2018; Gopal et al., 2019), second, several other likely and mechanistically plausible targets have been identified, and finally, results demonstrate that a PanD loss-of-function auxotrophic Mtb mutant is still vulnerable to POA when supplemented with PantSH, *i.e.*, when the deleterious effects due to defects in Pan synthesis (and therefore CoA biosynthesis) have been alleviated (Dillon et al., 2014; Anthony et al., 2018; Gopal et al., 2019).

#### Assessing PanD as a Drug Target

The targetTB *in silico* target identification pipeline did not identify *Mt*PanD as a high confidence target (Raman et al., 2008). It was excluded from the list of targets as the structural assessment of targetability indicated likely structural homology between its identified binding pockets and those identified in the human proteome, suggesting that the design of selective inhibitors might be difficult to achieve. However, as this analysis was done using generally applied algorithms, an investigation of the known *Mt*PanD binding sites might indicate otherwise. In addition, no targeted knockdown of *panD* has been conducted to date—the main reason being that the adjacent *panC* and *panD* genes are positioned so close to one another that it would not be possible to effectively reduce the translation of one without also affecting the other. As such, it is likely that the results of the targeted knockdown of *panC* at least partially reflects the impact on knockdown of *panD* as well (Abrahams et al., 2012).

However, the combination of the lower virulence of the double Δ*panCD* mutant, and the higher survival rates of mice infected with this strain (Sambandamurthy et al., 2002), together with recent findings that tie PZA’s antiTB MoA to effects related to *Mt*PanD, make a strong case for this enzyme as a promising candidate for further drug design efforts (Gopal et al., 2019; Gopal et al., 2020). Moreover, western blot and shotgun proteomic analyses indicate that the intrabacterial concentration of *Mt*PanD is low compared to the other Pan and CoA biosynthetic enzymes (Gopal et al., 2020).

### PanE – Ketopantoate Reductase (KPR)

#### Enzyme Structure and Mechanism

PanE, the ketopantoate reductase (KPR) enzyme specifically responsible for reduction of ketopantoate **9** to d-pantoate **5** (**Figure 1A**), long remained elusive. Its identification was complicated, in part, due to several other enzymes being capable of catalyzing this reduction (Webb et al., 2004). Ketopantolactone reductase is one such example, along with enzymes isolated from both *Candida parapsilosis* and spinach chloroplasts (King et al., 1974; Hata et al., 1989; Julliard, 1994; Webb et al., 2004). The first sample with KPR activity was purified in 1988 (Shimizu et al., 1988), and the structure of the apo enzyme from *E. coli* was solved in 2001 (Matak-Vinković et al., 2001). Crystal structures of *E. coli* PanE with the NADP^+^ cofactor bound, and the ternary complex with NADP^+^ and **5**, have since been characterized (Lobley et al., 2005; Ciulli et al., 2007). The PanE enzymes of *S. enterica* Typhimurium, *Stenotrophomonas maltophilia* and *E. coli* have been studied (Webb et al., 2004). Kinetic parameters have been rigorously investigated for *E. coli* PanE, leading to an ordered sequential kinetic mechanism being proposed for the enzyme (Zheng and Blanchard, 2000a; Zheng and Blanchard, 2000b; Zheng and Blanchard, 2003).

To date, there is very little information available for the Mtb PanE homologue (*Mt*PanE). The activity of the protein expressed by the putative *panE* gene (Rv2573) has not been experimentally verified, although its crystal structure bound to NADP^+^ and oxamate has been solved (PDB ID: 4OL9).

#### PanE Inhibitors

The native cofactor has been used as a template for fragment-based targeted binding to identify hot spots within the *E. coli* PanE active site along with the specific regions required for recognition within the cofactor site. This data is valuable in view of future inhibitor design, but no actual inhibitors have been identified as yet (Ciulli et al., 2006; Ciulli et al., 2007). It is also unclear if this information will translate to the *Mt*PanE protein.

#### Assessing PanE as a Drug Target

The targetTB comprehensive *in silico* target identification pipeline identified *Mt*PanE as a high confidence target (Raman et al., 2008). Moreover, the levels determined for *Mt*PanE in an Mtb proteomic analysis were the lowest of all the Pan and CoA enzymes that could be detected (*Mt*PanD was excluded from this analysis) (Schubert et al., 2013). However, the *panE* gene is non-essential in Mtb by Himar-1 transposon mutagenesis (Sassetti et al., 2003; Griffin et al., 2011; DeJesus et al., 2017), most likely due to the ability of other enzymes to perform the reduction at sufficient levels to provide the needs for Pan biosynthesis. Moreover, Mtb conditional knockdown mutants in *panE* are refractory to growth attenuation (Evans et al., 2016). Taken together, this points to *Mt*PanE possibly being a poor target in Mtb.

## Targeting Coenzyme A Biosynthesis

As indicated in the previous section, most bacteria—including Mtb—are able to synthesize their own Pan *de novo*, but can also source it from the environment. Successfully targeting the Pan biosynthetic enzymes, therefore, depends on a range of factors as highlighted in the description of each respective enzyme. In contrast, all organisms (barring a few rare exceptions) must obtain CoA through biosynthesis from Pan using their own biosynthetic machinery. This becomes especially important during cell division, as the cellular CoA pool otherwise remains relatively stable (Leonardi et al., 2005; Strauss, 2010). As such, the CoA biosynthetic enzymes of Mtb have been regarded as promising targets for antitubercular drug development, with much energy being expended by both industry and academic groups to identify new inhibitors of these enzymes, especially through a number of HTS efforts. The outcome of these and other studies are detailed in the following section.

### CoaA – Pantothenate Kinase (PanK)

#### PanK Enzyme Structure and Mechanism

Pantothenate kinase (PanK) catalyzes the ATP-dependent phosphorylation of Pan **2**, functioning as an entry point to the CoA biosynthesis pathway (**Figure 1B**). Interestingly, Mtb is one of a few bacteria (including *Bacillus subtilis*) with two different, structurally unrelated PanK enzymes (Strauss, 2010). The first, *Mt*PanK, is encoded by the *coaA* gene (Rv1092c) and belongs to the type I PanKs that are typified by the *E. coli* enzyme (*Ec*PanK). The other, *Mt*CoaX, is a type III PanK encoded by *coaX* (Rv3603c), which is found in an open reading frame with *panD* and *panC*. However, unlike other well-characterized type III PanKs such as the enzymes from *Pseudomonas aeruginosa* and *Bacillus anthracis*, the *Mt*CoaX enzyme seems to be inactive (Awasthy et al., 2010), and very little is known about its role (if any) in Mtb physiology. Consequently, most of the published studies have focused on investigating the structure and mechanism of the type I *Mt*PanK, with crystal structures of the protein in complex with substrates, products, and a variety of inhibitors (including one with CoA, the natural feedback inhibitor) being available (Das et al., 2005; Das et al., 2006; Chetnani et al., 2009; Chetnani et al., 2010; Chetnani et al., 2011; Paul et al., 2017). The *Mt*PanK enzyme is a homodimeric P-loop kinase with a central, seven-stranded β sheet, six of which are parallel and the seventh anti-parallel, flanked on either side by α-helices (Das et al., 2005; Das et al., 2006). Unique among described type I PanKs, the enzyme is able to use both GTP and ATP as phosphate donors with near identical efficiency (Chetnani et al., 2010). Comparison of the *Ec*PanK and *Mt*PanK structures indicates that this ability relates to small differences in the nucleotide binding sites of the enzymes. It has been hypothesized that this is an adaptation related to the ability of Mtb to enter a latent state during infection (Chetnani et al., 2010).

The *Mt*PanK kinetic parameters have been reported using both a standard pyruvate kinase/lactate dehydrogenase (PK/LDH) based kinase assay that couples the formation of ADP to the oxidation of NADH, as well as using isothermal titration calorimetry (ITC) (Kumar et al., 2007; Chetnani et al., 2009; Venkatraman et al., 2012). Interestingly, while both methods give similar *K*_M_ values for ATP (~100–180 μM), the reported *k*_cat_ values differ more than an order of magnitude depending on the method and the report: using the coupled assay, values of 0.61 s^–1^ and 5 ± 2 s^–1^ were reported, while using the ITC-determination values of ~0.4–0.6 s^–1^ were obtained. Although there are also other small differences in the conditions between the assays (*e.g.*, pH), it is unlikely that these could account for such a large divergence. One of the studies that used the ITC method also determined a *k*_cat_ value for *Ec*PanK of ~1.14 s^–1^ (Chetnani et al., 2009); since this is nearly identical to the value of 1.16 s^–1^ reported using the coupled assay (de Villiers et al., 2014), it is likely that the value determined by ITC and the lower value determined using the coupled assay more closely reflects the enzyme’s actual activity. *Mt*PanK’s reported *K*_M_ for Pan is also higher than that of *Ec*PanK (100–395 μM vs. ~21 μM); this suggests that *Mt*PanK is much less efficient than its *E. coli* counterpart (Kumar et al., 2007; De Villiers et al., 2010; Venkatraman et al., 2012).

**Figure 6:**
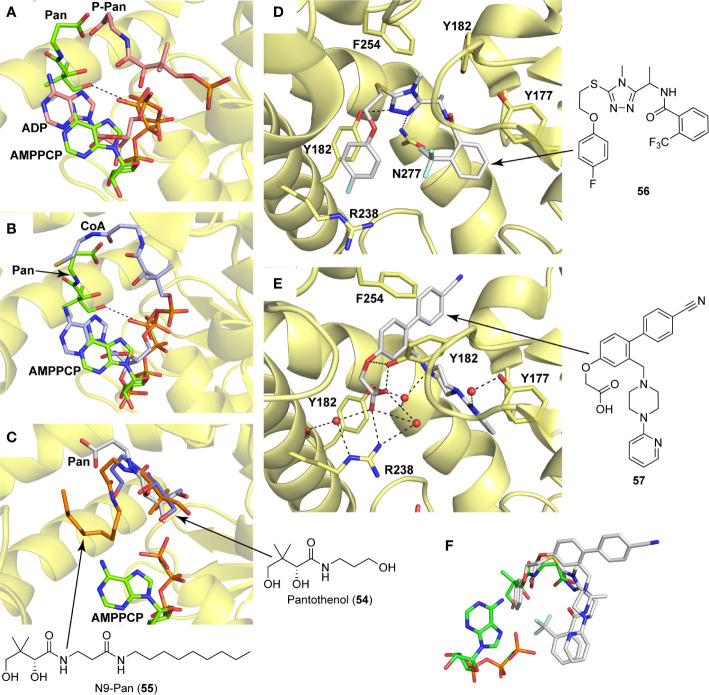
PanK structure and substrate and inhibitor interactions. **(A)** Overlay of the structures of *Mt*PanK bound to Pan (**2**) and the ATP analog AMPPCP (stick structures with carbon atoms colored light green) (PDB: 2ZSE), and of *Mt*PanK bound to P-Pan (**10**) and ADP (stick structures with carbon atoms colored salmon) (PDB: 2ZSA), representing the initiation and end complexes respectively. The dashed line indicates the path of phosphoryl transfer. **(B)** Overlay of the structures of *Mt*PanK bound to Pan (**2**) and AMPPCP (stick structures with carbon atoms colored light green) (PDB: 2ZSE), and of *Mt*PanK bound to CoA (**1**) (stick structure with carbon atoms colored light blue) (PDB: 2ZSD), showing how the binding of CoA competitively inhibits catalysis. **(C)** Overlay of the structures of *Mt*PanK bound to Pan (**2**) (stick structure with carbon atoms colored gray) (PDB: 3AVO), pantothenol (**54**) (stick structure with carbon atoms colored blue) (PDB: 3AVP), and N9-Pan (**55**) (stick structure with carbon atoms colored orange) (PDB: 3AVQ), indicating the similar binding poses for Pan and its analogues. **(D)** Structure of *Mt*PanK bound to the triazole inhibitor **56** (PDB: 4BFS) with the dashed lines showing the polar contacts between the ligand and protein residues. Key active sites residues are shown as sticks and labeled. **(E)** Structure of *Mt*PanK bound to the biaryl acetic acid inhibitor **57** (PDB: 4BFY) with the dashed lines showing the polar contacts between the ligand, protein residues and solvent (shown as red spheres). Key active sites residues are shown as sticks and labeled. **(F)** Overlay of the structures of Pan (**2**) and AMPPCP as bound in the initiation complex (panel **a**) with those of inhibitors **56** and **57** (panels **D, E**), showing that the latter mainly occupy the Pan binding site.

A detailed structural analysis by Vijayan and co-workers has suggested that, unlike what is observed for its homologue *Ec*PanK, the *Mt*PanK active site does not undergo significant structural changes during substrate binding and catalysis (Das et al., 2006; Chetnani et al., 2009; Chetnani et al., 2010; Chetnani et al., 2011; Paul et al., 2017). Instead, the substrates and products change positions within a preformed binding site which maintains its structure during catalysis (Chetnani et al., 2009; Chetnani et al., 2010; Chetnani et al., 2011). The changes in binding positions seem to occur due to higher affinity binding sites becoming available as both Pan **2** and the nucleotide triphosphate (NTP) bind, and as catalysis occurs. Specifically, an ordered mechanism is proposed in which the NTP binds first in a site that partially overlaps with that of Pan **2** in the initiation complex. The subsequent binding of Pan and its movement into the initiation complex displaces the NTP from its initial binding position (**Figure 6A**). This causes a change in the NTP’s conformation from extended to closed, with the γ-phosphate directly aligned to the 4′-OH group of Pan within the initiation complex (Chetnani et al., 2009; Chetnani et al., 2011). Once Pan **2** is converted to P-Pan **10**, the latter moves back to the initial Pan binding site, opening up the initial NTP binding site (which is shared with the NDP binding site) and allowing the catalytic cycle to restart (**Figure 6A**). The binding sites in the initiation complex are only accessed once both substrates have bound; this access is mediated by the conformation of the side chain of Arg238 which changes upon binding of Pan. In the initiation complex, the guanidium group of Arg238 bridges the two substrates (Chetnani et al., 2009; Chetnani et al., 2011).

An important feature of most type I PanK enzymes is that the final product of the pathway, CoA **1**, also binds to the enzyme in such a manner that both the Pan and NTP binding sites of the initiation complex are blocked (**Figure 6B**) (Chetnani et al., 2009). Consequently, CoA can function as a feedback inhibitor of PanK (Leonardi et al., 2005; Strauss, 2010). Interestingly, unlike *Ec*PanK, heterologously expressed *Mt*PanK purifies with CoA bound, and the ligand could only be removed through repeated dialysis against citrate buffer. Subsequent crystallographic analysis indicated that this was due to CoA being displaced by two citrate molecules (Chetnani et al., 2009). Analysis of the CoA binding thermodynamics through isothermal titration calorimetry (ITC) confirmed that *Mt*PanK has a higher affinity for CoA (*K*_d_ 4.7 μM) than does *Ec*PanK (*K*_d_ 6.3 μM), and is purified with ~65% of binding sites occupied with CoA [compared to ~16% for *Ec*PanK (Chetnani et al., 2009)]. Kinetic analysis determined the *K*_i_ for the inhibition by CoA to be 2 μM. However, the importance of CoA’s binding to *Mt*PanK has not been studied in a physiologically relevant context as yet; it therefore remains uncertain what impact this has on CoA biosynthesis in the cell, and, as such, on the development of PanK-targeting inhibitors.

#### Alternate Substrates and Metabolic Activation of Antimetabolites

Apart from being the first enzyme of both the *de novo* and salvage pathways for CoA biosynthesis (**Figures 1B, C**), PanK also acts as the gateway to the metabolic activation of Pan antimetabolites such as pantothenol (**54**) and the pantothenamides (**Figure 2C**), both of which act as prodrugs that only exert their inhibitory effect once phosphorylated (Kumar et al., 2007; Moolman et al., 2014). The potency of these compounds therefore largely depends on two factors: the relative specificity constant of PanK for conversion of the antimetabolite compared to Pan **2**, and the relative intracellular concentration of Pan and the antimetabolite. While a detailed comparative kinetic analysis of *Mt*PanK’s ability to accept a range of Pan analogues has not been performed as has been done for other bacterial PanKs (de Villiers et al., 2014), structural analysis of binary complexes of the enzyme bound to Pan (**2**), pantothenol (**54**) and *N*-nonyl pantothenamide (N9-Pan, **55**) has been performed (**Figure 6C**) (Chetnani et al., 2011). This indicates that in the absence of the NTP, all three compounds bind in a similar manner, occupying the P-Pan (**10**) binding site of the end complex (**Figure 6A**). This is the site where binding is proposed to first occur before these substrates move to the binding site of the initiation complex (see above). Combined with evidence that *Mt*PanK phosphorylates both pantothenol (**54**) and N9-Pan (**55**) (in the former case with ~¼ of the efficiency compared to that seen for Pan) (Kumar et al., 2007; Chetnani et al., 2011), this suggests that it, like other type I PanKs, is able to accept a wide range of Pan analogues as alternate substrates.

#### PanK Inhibitors Developed Through Rational Design

There have not been any rationally (structure-based) designed drug candidates reported for *Mt*PanK yet. Rational drug development has mainly been pursued after the initial hits have been identified.

#### PanK Inhibitors Identified by Screening

An HTS and several smaller screening studies have focused primarily on identifying inhibitors of the enzyme, while others have identified *Mt*PanK as the (or one of the) target(s) of specific drug candidates that had previously demonstrated whole cell activity.

Venkatraman et al. (2012) adapted the standard PK/LDH-based coupled kinase assay for HTS (Brand and Strauss, 2005). Inhibitors were screened at 25 µM while ATP and Pan were used at the concentration of the *K*_M_ values determined for these compounds using the coupled kinase assay (as detailed in ***Section 3.1.1***). Two libraries were screened, with the first (70K compounds) focused on triazole and quinoline scaffolds, while the second (~1 million compounds) represented a more diverse range of molecules. The scaffolds of interest that were identified included triazoles, thiazoles, quinoline carboxamides and biaryl acetic acids. A high-throughput method of determining the mechanism of inhibition was designed and verified, with the triazoles and quinolones being found to be ATP competitive, while the biaryl acetic acid compounds showed mixed non-competitive inhibition (Venkatraman et al., 2012; Reddy et al., 2014). IC_50_ values were determined alongside inhibition constants for both the enzyme and the enzyme-substrate complex. The initial hits were further improved, resulting in the quinoline carboxamide scaffolds becoming quinolone amides. Detailed structure-activity relationships (SARs) of the identified classes of inhibitors were also investigated based on the structural characterization of the exact binding interactions within the active pocket for the triazole and biaryl classes (Björkelid et al., 2013; Reddy et al., 2014). This shows that the triazoles (such as compound **56**) (**Figure 6D**) and the biaryl acetic acids (such as compound **57**) (**Figure 6E**) occupy a similar binding site in the active site, but through different protein–ligand interactions. Interestingly, both the triazoles and biaryl acetic acids mainly overlap with the Pan binding site of the initiation complex (**Figure 6F**), even though the former was kinetically shown to be an ATP competitive inhibitor. This observation may be explained by the inhibitors causing Arg238—the residue acting as gatekeeper in the movement of ligands in the active site—to take on different conformations (compare **Figures 6D, E**).

The optimized set of inhibitors showed submicromolar IC_50_ values (**Table 6**, **58**–**61**). However, since cellular ATP levels can be up to ten times higher than *Mt*PanK’s determined *K*_M_ for ATP (~120 μM), IC_50_ values were also determined at 50×*K*_M_ (6 mM) to confirm maintained potency and inhibition mechanism (Buchholz et al., 2001; Reddy et al., 2014). Hit potency was significantly optimized to obtain representatives of the triazoles, quinolones and biaryl acetic acids with IC_50_ values of 80 nM (**58**), 210 nM (**59**) and 22 nM (**61**), respectively. Unfortunately, the ATP competitive inhibitors, namely the triazoles and quinolones, did not display whole cell activity despite the optimized potency (Reddy et al., 2014). They did, however, show growth inhibition against *coaA* knockdown mutants with reduced expression of *Mt*PanK, indicating that the compounds acted on target, and that the lack of whole cell activity was not due to poor permeability. Ester derivatives with IC_50_ values lower than 1 µM in this class showed whole-cell activity ranging between 4 µg/mL (**60**) and 16 µg/mL. Carboxylic acid derivatives of this class, though very potent, did not show any whole cell activity, suggesting that they could not cross the cell membrane (Reddy et al., 2014).

**Table 6:** Structures and inhibition characteristics of *Mt*PanK inhibitors identified through screening of natural products and optimization of high throughput hits^a^.

Entry	Structure	IC_50_ (µM)	MIC (µg/mL)
**56**	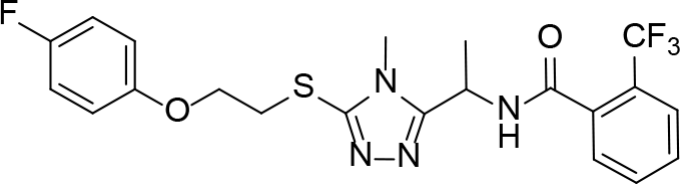	0.08	>64
**57**	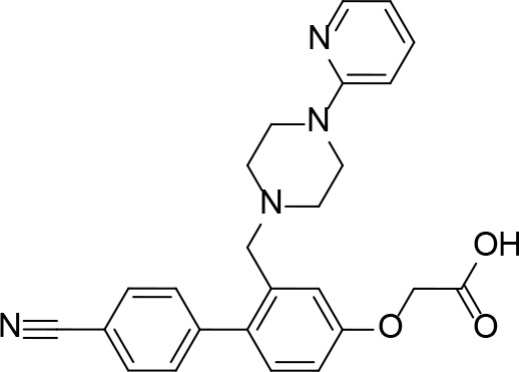	0.2	32
**58**	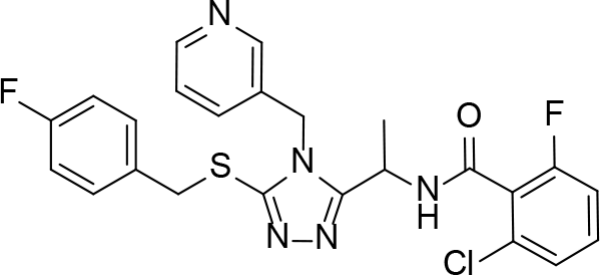	0.08	>64
**59**	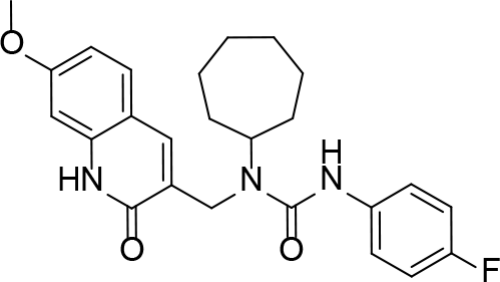	0.21	>64
**60**	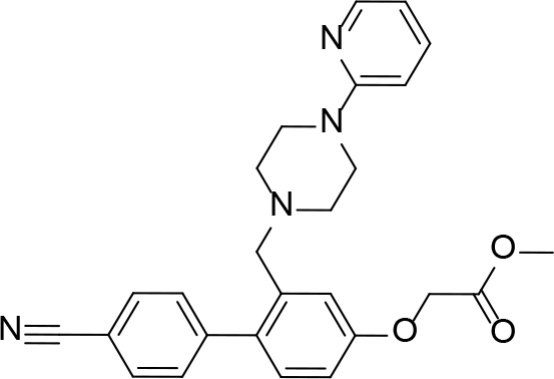	0.89	4
**61**	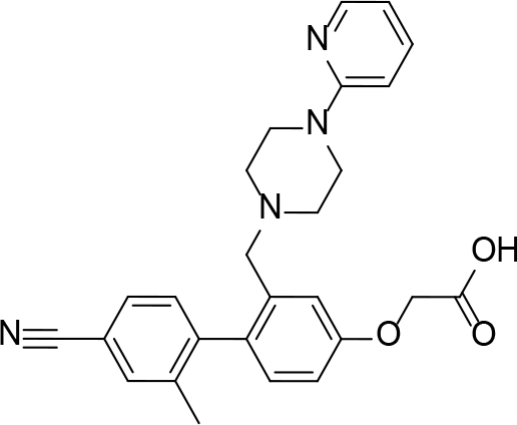	0.02	32
**62**	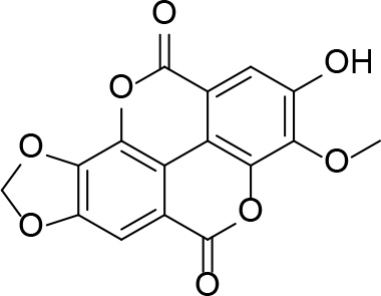	Purely computational	
**63**	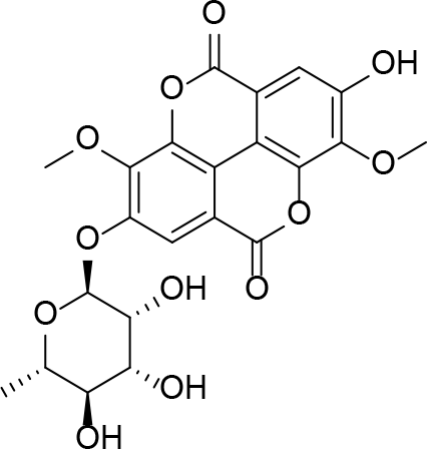	Purely computational	
**64**	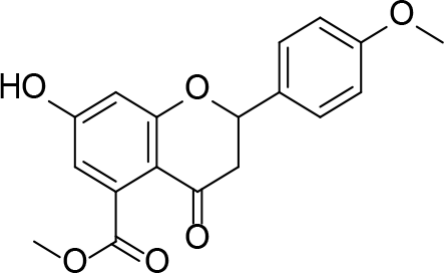	39.38	>30
**65**	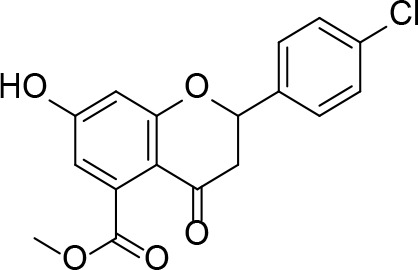	25.34	27.26
**66**	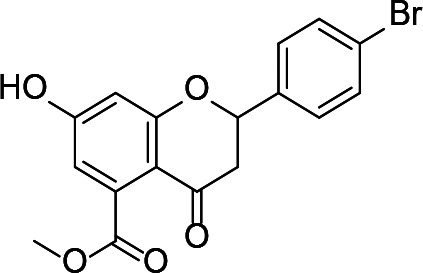	29.96	>30
**67**	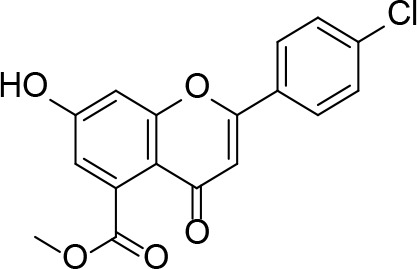	53.13	28.90

^a^Compounds **58**–**61** are optimized hit compounds identified by (Venkatraman et al., 2012; Björkelid et al., 2013; Reddy et al., 2014) by using a PK/LD coupled kinase assay. Compounds **56**, **58** and **59** are ATP competitive inhibitors while compounds **57**, **60** and **61** are mixed noncompetitive. Compounds **62–67** are natural products and their analogues investigated by (Shilpi, 2009; Shilpi et al., 2015) and (Puranik et al., 2018), respectively, with the latter being identified through an XRMA assay. All values are shown as reported in the original studies.

Two studies investigated natural product-derived inhibitors active against *Mt*PanK. Both were investigating inhibitors for essential enzymes involved in cell wall biosynthesis, particularly through fatty acid biosynthesis (Shilpi et al., 2015; Puranik et al., 2018). The authors investigated inhibition against *Mt*PanK primarily because of its potential to activate antimetabolites. Shilpi et al. (2015), in a purely computational study, investigated ellagic acid and its α-l-rhamnose glycoside (**Table 6**, **62** and **63**), which are known to target mycolic acid biosynthesis. The two derivatives tested had previously shown some activity against *Mycobacterium aurum in vitro* and, upon docking into the Pan binding site of *Mt*PanK, had similar docking scores and interacted with a greater number of residues as compared to one of the triazole inhibitors identified in the earlier reported HTS study (Björkelid et al., 2013). Similarly, rugosaflavonoid derivatives were investigated for their potential *Mt*PanK inhibitors through an *in silico* docking study (Puranik et al., 2018). The active site of *Mt*PanK was set up for docking through receptor grid generation from a crystal structure with the bound cofactor. Four compounds were identified that showed docking scores and active site interactions similar to those seen with isoniazid and quercetin, which were used as reference compounds with known biological activity (albeit not necessarily targeting PanK) (**Table 6**, **64**–**67**). An XTT Reduction Menadione Assay (XRMA) was used to determine these compounds’ potency as whole cell inhibitors with the obtained IC_50_ values ranging between 8.43 µg/mL and 17.57 µg/mL. However, no confirmation of on-target activity was performed.

Lastly, Chiarelli et al. (2018) determined that two prodrugs that were previously shown to inhibit the CTP synthetase PyrG, also target *Mt*PanK. The compounds are activated by the monooxygenase EthA, which is mainly known for activating other antitubercular drugs such as ethionamide (Baulard et al., 2000; DeBarber et al., 2000). The inhibitors (**Table 7**, **68** – **71**) show surprising structural similarities to the biaryl acetic acid and thiazole scaffolds previously identified for optimization by Venkatraman and co-workers, as discussed above (Venkatraman et al., 2012; Björkelid et al., 2013). Inhibition of *Mt*PanK was measured by means of the PK/LDH coupled kinase assay, with a *K*_i_ of 22.9 ± 1.3 µM being determined for the active metabolite **71** (**Table 7**). The compounds were shown to inhibit *Mt*PanK by binding to the ATP pocket, as is seen for PyrG. As multitargeting inhibitors have a lower chance of generating resistant strains, previous libraries used for identifying PyrG inhibitors (GSK TB-set and Collaborative Drug Discovery (CDD) database) were rescreened against *Mt*PanK and several additional inhibitors were identified through both *in silico* and enzymatic methods. GSK1570606A, GSK920684A, GSK735826A (**68**) all had high docking scores but only **68** was active with a *K*_i_ value of 65.3 ± 4.3 µM (**Table 7**). CDD-934506 (**Table 7**, **69**) was identified with moderate activity against *Mt*PanK (IC_50_ of 40 µM), though it was inactive against *Mt*PyrG.

**Table 7:** Structures and inhibition characteristics of *Mt*PanK inhibitors identified through resistance mechanisms^a^.

Entry	Structure	IC_50_ (µM)	*K*_i_ (µM)	MIC (µM)
**68**	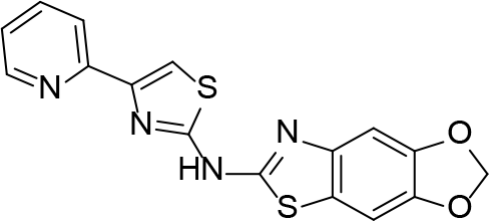	70	65.3	2.7
**69**	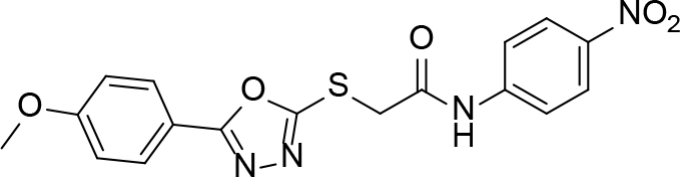	40	–	0.87
**70**	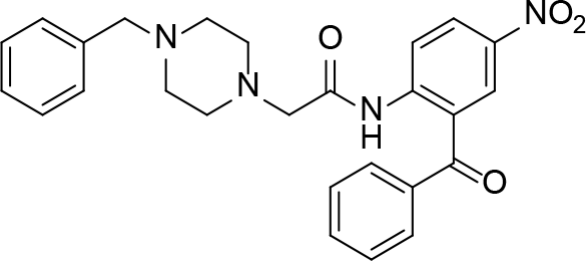	250	–	4.39
**71**	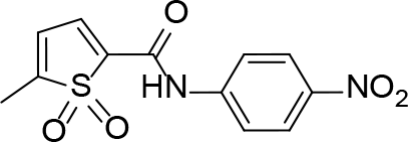	29.3	22.9	–

^a^Compounds **68**–**71** were identified by (Chiarelli et al., 2018) by means of a PK/LD coupled kinase assay; all are competitive inhibitors. All values are shown as reported in the original studies.

#### Assessing PanK as a Drug Target

The successes achieved to date in developing small molecule inhibitors of *Mt*PanK with submicromolar potency suggests that it is a highly tractable target. However, the failure of these inhibitors to show whole cell activity led to the investigation of this enzyme’s vulnerability in a physiological context. Subsequent targeted knockdown studies indeed seemed to indicate very poor vulnerability, with cells growing normally in liquid media even when intracellular levels of *Mt*PanK could not be detected by Western blotting (Reddy et al., 2014). This result was corroborated by the finding of Evans et al. (2016) who showed that even under conditions of maximal silencing of *coaA* expression, no growth phenotype could be observed. *Mt*PanK was nonetheless identified as a potentially good target in the targetTB target identification pipeline (Raman et al., 2008).

The basis for the apparent low vulnerability of *Mt*PanK, and the subsequent lack of success in translating potent *Mt*PanK inhibitors into whole cell inhibitors, remains unclear. One potential explanation that was considered was that the type III PanK encoded by the *coaX* gene could compensate for any inhibition of *Mt*PanK. However, under *in vitro* assay conditions, the enzyme encoded by *coaX* was found to be non-essential and later confirmed to be non-functional, both *in vitro* and *in vivo*, even though it was still expressed in the organism (Awasthy et al., 2010). An alternative reason for the poor performance of *Mt*PanK could be related to the control of its activity by CoA, which is an aspect that remains unexplored.

Taken together, the results in developing *Mt*Pank inhibitors to date suggest that, until we have a better understanding of this enzyme’s importance in the CoA pathway, its main role in TB inhibitor drug discovery may be limited to the activation of CoA antimetabolites.

### CoaBC – Phosphopantothenoylcysteine Synthetase (PPCS)/Phosphopantothenoylcysteine Decarboxylase (PPCDC)

#### CoaBC Enzyme Structure and Mechanism

CoaBC, a bifunctional bacterial protein, harbors the enzymatic activities of both PPCS and PPCDC, these being the second and third steps in the biosynthesis of CoA (Strauss, 2010). PPCS (also known as CoaB) catalyzes the Mg^2+^-dependent coupling of P-Pan (**10**) and cysteine in two steps: first, cytidylation of P-Pan with CTP to form P-Pan-CMP as activated intermediate, and second nucleophilic displacement of cytidine monophosphate (CMP) from the intermediate by l-cysteine to form P-PanCys (**11**). P-PanCys subsequently undergoes decarboxylation of its cysteine moiety by PPCDC (or CoaC) to yield the CoaBC product, P-PantSH (**7**).

Bacterial (Type I) PPCS enzymes, exclusively utilize CTP for activation of the P-Pan carboxylate moiety for acyl transfer, whereas PPCS enzymes in eukaryotes (Type II PPCS) usually prefer to use ATP (Strauss, 2010). Additionally, the fusion of PPCS to PPCDC in the single bifunctional CoaBC protein is an occurrence restricted to bacteria, while the eukaryotic PPCS and PPCDC enzymes are generally expressed by two different genes as separate monofunctional proteins. The Mtb enzyme (*Mt*CoaBC) is no different, and is a bifunctional protein encoded by the *coaBC* gene (Rv1391) like its other bacterial counterparts.

**Figure 7:**
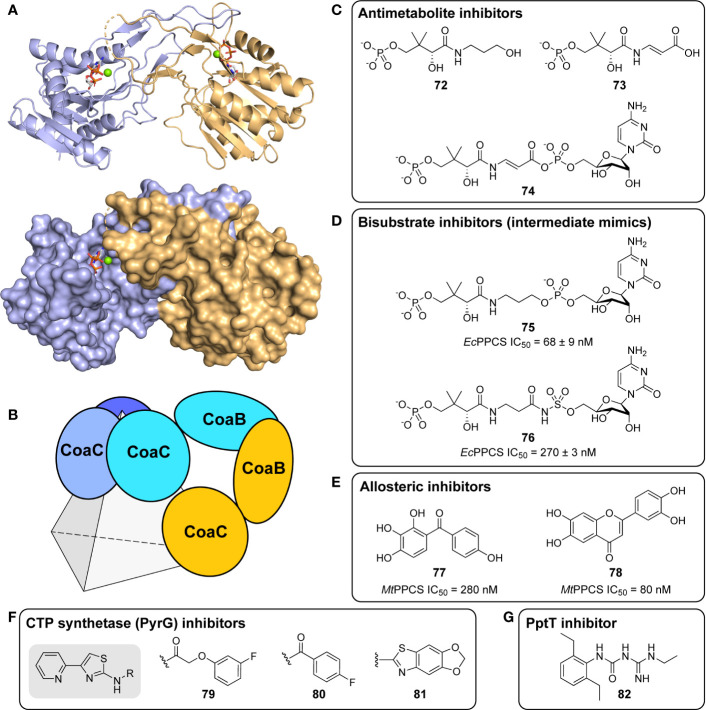
CoaBC structure and inhibitors. **(A)** Structure of *Ms*PPCS, *i.e.*, the CoaB domain of the *M. smegmatis* CoaBC (PDB: 4QJI), showing the dimer interaction in the cartoon and the active site pocket with CTP and Mg^2+^ bound in the surface model. The two subunits are shown in different colors. **(B)** Schematic representation of the dodecameric *Ms*CoaBC structure (Mendes et al., 2019), which takes on the shape of a tetrahedron, with the CoaB domains forming dimers at its six edges, and the CoaC domains forming trimers at its four vertices. In this representation only two CoaBC proteins (in cyan and orange) are shown to highlight the CoaBC interaction, with one CoaC trimer shown at the top vertex. **(C)** Structures of antimetabolite inhibitors of the PPCS activity of CoaBC proteins. 4′-Phosphopantothenol (**72**) and 4′-phospho-CJ-15,801 (**73**) are formed through the action of PanK enzymes on the corresponding Pan analogues, with **73** being transformed *in situ* into the nonreactive reaction intermediate **74**, that is a potent PPCS inhibitor. **(D)** Nonreactive bisubstrate inhibitors (structural mimics of the acyl nucleotide intermediate of PPCS) of several bacterial PPCS enzymes (Patrone et al., 2009). **(E)** Structures of allosteric inhibitors of the PPCS activity of *Mt*CoaBC discovered through an HTS (Mendes et al., 2019). **(F)** Structures of three potent Mtb CTP synthetase (PyrG) inhibitors from screening a GlaxoSmithKline library which are proposed to also affect *Mt*CoaBC (Esposito et al., 2017). **(G)** Structure of compound 8918 (**82**), an inhibitor of Mtb phosphopantetheinyl transferase (PptT) that also shows pleotropic effects on CoA biosynthetic intermediate levels. It is proposed that this occurs through inhibition of *Mt*CoaBC, although direct evidence of this remains lacking (Ballinger et al., 2019).

The crystal structures of *E. coli* PPCS (CoaB domain of the *E. coli* CoaBC protein) in complex with several ligands (Stanitzek et al., 2004), human PPCS (Manoj et al., 2003), yeast PPCS (Zheng et al., 2019) and human PPCDC (Manoj and Ealick, 2003) have been solved and analyzed, though the three-dimensional structures of the Mtb CoaBC (*Mt*CoaBC) or its constituent domains remain unknown. However, the coordinates of the structure of the *M. smegmatis* PPCS (*Ms*PPCS), i.e. the CoaB domain of the *M. smegmatis* CoaBC (*Ms*CoaBC) was previously deposited (PDB: 4QJI). This structure clearly shows the dimeric nature and subunit interaction of the CoaB domains, as well as the active site pocket with CTP bound (**Figure 7A**). Recently a preprint describing the structures of both the full *Ms*CoaBC and its CoaB domain was published (Mendes et al., 2019). The solution of a bacterial CoaBC crystal structure at 2.5 Å resolution is an important achievement, as it provides the first glimpse of the overall shape and structure of this multimeric protein. The structure shows that the protein is a dodecamer that takes on the shape of a tetrahedron, with the CoaB domains forming dimers at its six edges, and the CoaC domains forming trimers at its four vertices (**Figure 7B**). The two domains are joined by a loop region that interacts closely with each of them. The active site of the PPCDC enzyme (CoaC domain) lies at the interface of adjacent trimers. Unlike the *Ec*PPCS which shows activity when expressed on its own as the CoaB domain, the *M. smegmatis* CoaB does not and requires stabilisation by the CoaC domain (Kupke, 2002; Chan et al., 2019; Mendes et al., 2019). The PPCS active site is at the dimer interface (dimerization takes place between the CoaB domains that point outward from the CoaC trimer, *i.e.*, adjacent trimers dimerize *via* CoaB domains) and is enclosed by a loop from an opposing protomer (Mendes et al., 2019). The authors confirmed that like the *Ms*CoaBC, the *Mt*CoaBC protein is a dodecamer as well, with the two proteins having molecular weights of 523 kDa and 537 kDa, respectively (Mendes et al., 2019).

#### CoaBC Inhibitors Formed Through Metabolic Activation of Antimetabolites

In 2007, it was first demonstrated that pantothenol (**54**) acts as an alternate substrate for *Mt*PanK, forming 4′-phosphopantothenol (**72**) (**Figure 7C**) (Kumar et al., 2007). This subsequently acts as a competitive inhibitor of CoaBC, preventing CoA biosynthesis from continuing. This was demonstrated by means of an activity analysis making use of an assay relying on the conversion of radiolabeled P-Pan (**10**). In the assay, varying amounts of inhibitor were incubated with the enzyme and substrates for 2 min, after which the reaction was stopped by heating denaturation and the products were separated *via* thin-layer chromatography (TLC). New spots were identified by ESI-MS and conversion was calculated by determining the amount of product formed using radiodensitometry analysis. In this manner, an IC_50_ value of 63 µM pantothenol was determined for inhibition of *Mt*CoaBC activity (Kumar et al., 2007). Based on these findings the authors postulate that pantothenol is likely to inhibit the growth of other bacteria using a similar mechanism of action (metabolic activation by PanK followed by inhibition of the PPCS activity of the CoaBC protein by 4′-phosphopantothenol).

A natural product analogue of Pan (**2**), CJ-15,801, was shown to exhibit selective, micromolar whole cell activity against drug resistant strains of *S. aureus*, whereafter it was identified as a tight-binding inhibitor of *Ec*PPCS and *Sa*CoaBC (Sugie et al., 2001; van der Westhuyzen et al., 2012). Its mechanism of inhibition was determined to proceed *via* phosphorylation of CJ-15,801 by *Sa*PanK (*i.e.*, metabolic activation) to form 4′-phospho-CJ-15,801 (**73**), and subsequent formation of the corresponding cytidylylated intermediate (**74**) by PPCS (**Figure 7C**). This intermediate was shown to be unreactive, and therefore exhibited excellent inhibition by acting as a structural mimic of the natural reaction intermediate, with *K*_i_ values of 164.3 nM for *Ec*PPCS and 13 nM for *Sa*CoaBC (van der Westhuyzen et al., 2012). The study demonstrated that CJ-15,801 is selective for *S. aureus* because of the unique substrate specificity of the type II *Sa*PanK; type I PanKs (including those of *E. coli* and Mtb) does not act on CJ-15,801, and therefore metabolic activation does not occur. However, it may be possible to circumvent this requirement for metabolic activation by preparing prodrug versions of the activated inhibitor **73** in which the polar phosphate and carboxylate groups are protected with labile esters groups. This strategy has been successfully used to convert the natural product fosmidomycin—an inhibitor of Mtb 1-deoxy-D-xylulose-5-phosphate reductoisomerase (DXR) with a polar phosphonate moiety—into cell-permeable whole cell inhibitors (Wang and Dowd, 2018).

#### CoaBC Inhibitors Developed Through Rational Design

To date, there have been no reports on any *Mt*CoaBC inhibitors developed by rational design approaches. This is likely due to the lack of an available crystal structure, and may likely change with the report of the structure of the *Ms*CoaBC protein. Nonreactive analogues of the 4′-phosphopantothenoyl-nucleotidylate intermediate formed during the PPCS reaction have been explored as inhibitors of *Enterococcus faecalis*, *Streptococcus pneumonia*, and *E. coli* PPCS enzymes (Patrone et al., 2009). A series of structural mimics of the intermediate were designed and synthesized. Significantly, compounds **75** and **76** (**Figure 7D**) were found to have up to 1000-fold and 740-fold selectivity, respectively, for bacterial PPCS over the human enzyme *in vitro*, both with IC_50_ values in the nanomolar range (Patrone et al., 2009). For these analyses, a continuous spectrophotometric assay was used that couples the production of pyrophosphate in the first step of the reaction to the oxidation of NADH through the intermediacy of six coupling enzymes. In this manner the inhibitors were identified as being tight-binding and non-competitive with CTP. Furthermore, **75** was shown to undergo both slow association and dissociation with the enzyme and was subsequently classified as a slow-onset inhibitor. This was supported by analysis of the progress curves that showed a time-dependent decrease in reaction rate and plots of *k*_obs_ against inhibitor concentration that were linear. Unfortunately, the compounds in this study failed to exhibit activity against bacterial growth, likely due to a lack of cell penetration because of the presence of the polar and ionized terminal phosphate group.

#### CoaBC Inhibitors Identified by Screening

As part of the recent report on the structures of the *M. smegmatis* CoaB and CoaBC proteins, an HTS of 215K compounds was performed against *Mt*CoaBC using an endpoint assay that determines the amount of pyrophosphate formed after two hours (Mendes et al., 2019). The most potent hits were characterized further by determining IC_50_ values using the EnzCheck coupled pyrophosphate assay. A search of commercially available analogues of the initial hits resulted in the identification of two compounds (hydroxylated diphenyl ketone **77** and chromone **78**, **Figure 7E**) with sub-micromolar IC_50_ values (280 and 80 nM, respectively). It should be noted that these assays were performed using 32 nM enzyme, *i.e.*, the determined IC_50_ values are less than 10-fold the enzyme concentration used. This would indicate tight-binding inhibition, and standard steady state kinetic analysis would not apply in such a case. These values should therefore be understood taking this into consideration.

Co-crystallization of *Ms*CoaBC in complex with CTP revealed that **77** binds in a deep cavity that links to the active site. This cavity, which is located at the interface of the CoaB dimer, is obstructed when the native P-Pan (**10**) substrate is not bound. It was further shown that two of these extended pockets are present in each CoaB dimer. The opening and closing of these allosteric binding sites are largely mediated by a highly conserved arginine residue, Arg207, which is hypothesized to move upon binding of cysteine and is known to be intimately involved in the conversion of P-Pan-CMP to P-PanCys (**11**). Further elucidation of these compounds’ mechanism of inhibition led to the hypothesis that the substrate-bound enzyme is stabilized upon binding of the inhibitors to the allosteric site as this locks the side chain of Arg207 in place, thus removing the enzyme from the catalytic cycle (Mendes et al., 2019). The inhibitors did not display whole cell activity, though some moderate to low activity was displayed in albumin-free media. It was suggested that the difference between the enzymatic and whole cell results could be due to albumin binding, efflux, low permeation or metabolization. In summary, this novel allosteric site located at the dimer interface provides the potential for inhibiting the PPCS activity of *Mt*CoaBC (Mendes et al., 2019).

In a fragment-based screen utilizing differential scanning fluorimetry and NMR, an aryl sulfonamide was identified as a potential inhibitor of *Ec*PPCS (Chan et al., 2019). Several simpler aromatic derivatives of this fragment were then synthesized and screened with native MS (ESI-MS from a non-denaturing solvent) that identified five hits. These results were further corroborated by means of a coupled enzymatic assay (using the EnzCheck pyrophosphate assay kit) that monitors the production of pyrophosphate in the presence of inhibitors. The inhibitors identified as hits by native MS were tested at 1 mM, and their relative amount of inhibition of PPCS activity was found to range between 29 and 92%. Inhibition values of those compounds not identified as hits in the native MS screen were below 29% (Chan et al., 2019).

Following the identification of the Mtb CTP synthetase PyrG as a new antiTB drug target, a GlaxoSmithKline compound library was used in an HTS study to identify new CTP synthetase inhibitors (Esposito et al., 2017). This resulted in the identification of a series of 4-(pyridine-2-yl) thiazole analogues with activity against Mtb PyrG. The three most active compounds exhibited low micromolar *K*_i_ values (**79**–**81**, **Figure 7F**). Additionally, these compounds demonstrated specificity for CTP synthetases, lacking activity against a panel of prokaryotic and eukaryotic kinases (Esposito et al., 2017). The authors proposed that these compounds would also impact the activity of *Mt*CoaBC due to its requirement of CTP for activity. While it was shown that the PyrG inhibitors decreased the incorporation of acetate into cells, indicating a pleotropic effect, no direct impact on *Mt*CoaBC activity was determined or demonstrated.

Recently, the amidino-urea 8919 (**82**) was discovered to inhibit phosphopantetheinyl transferase (PptT) (**Figure 7G**). PptT is the enzyme responsible for post-translational activation of the *apo*-acyl carrier protein (*apo*-ACP) into its active *holo* form through the transferal of CoA’s P-PantSH moiety of a conserved Ser residue in the protein. The authors proposed **82** might also inhibit *Mt*CoaBC based on the impact of the inhibitor on Mtb’s metabolism through a metabolomics analysis (Ballinger et al., 2019). However, similar to the PyrG inhibitors, direct evidence of inhibition of any of *Mt*CoaBC’s activities by **82** remains lacking.

Finally, a team from the University of Michigan (UM) reported the results of an HTS performed to identify inhibitors of bacterial PPCS enzymes (Gómez-Rodríguez et al., 2020). They used the malachite green end point assay for pyrophosphate formation to screen the UM Center for Chemical Genomics natural product extract (NPE) library; specifically, 11,000 NPEs derived from marine microbes were screened. After counter-screening, 22 strains producing inhibitors of the *S. pneumoniae* PPCS (*Sp*PPCS) were identified. Fractionation and structural characterization identified the adipostatins, natural products that share a common alkylresorcinol structure, as the active PPCS inhibitors in the NPEs. Subsequent anti-microbial growth assays identified adipostatin E as being the most potent, with an IC_50_ value of 930 nM against *Sp*PPCS and whole cell activity against five Gram-positive pathogens, with IC_50_ values between 3.4 and 15.6 μM. Gram-negative bacteria was resistant to inhibition. Activity against mycobacteria was not tested.

#### PPCDC Inhibitors Developed Through Rational Design or Screening

No inhibitor has been reported to act on the PPCDC activity of *Mt*CoaBC to date. The only known inhibitor of any PPCDC activity is a cyclopropyl-substituted 4′-phospho-*N*-(1-mercaptomethyl-cyclopropyl)-pantothenamide (P-PanΔSH) that was shown to act as a mechanism-based inhibitor of the human PPCS enzyme (Strauss et al., 2004).

#### Assessing CoaBC as a Drug Target

As described above, Evans et al. (2016) created a panel of conditional knockdown mutants in genes encoding six potential targets within the CoA pathway (PanB, PanE, PanK, CoaBC, CoaD, and CoaE) and used to assess the viability of Mtb. From this seminal study, it was shown that depletion of *coaBC*, both *in vitro* and *in vivo* in infected mice, was the only bactericidal target in the CoA biosynthetic pathway. This important work inspires studies examining CoaBC as a novel drug target. Although limited progress has been made on the discovery of inhibitors to date, the significant differences between bacterial and mammalian enzymes, the recent discovery of an allosteric site that remains unexplored by rational drug design, and the importance of CoaBC to Mtb viability *in vivo* all ensure that CoaBC remains an important target for antimycobacterial drug development.

### CoaD – Phosphopantetheine Adenylyltransferase (PPAT)

#### PPAT Enzyme Structure and Mechanism

Phosphopantetheine adenylyltransferase (PPAT) catalyzes the reversible Mg^2+^-dependent adenylylation of P-PantSH (**7**) to form DePCoA (**12**) and pyrophosphate. The *E. coli* enzyme has been shown to tightly bind CoA, which results in inhibition of its activity (Miller et al., 2010); a similar result is obtained for the Mtb enzyme (Wubben and Mesecar, 2010). As such, PPAT has been proposed as a second point of feedback control by CoA on the pathway (after PanK) (Jackowski and Rock, 1984; Strauss, 2010). A wealth of detailed biochemical and structural studies has been reported on PPAT from *M. tuberculosis* (*Mt*PPAT) encoded by the *coaD* gene (Rv2965c), providing detailed characterization of the active site and insight into the inhibitory mechanism of the enzyme. The structures include: the three-dimensional structure of apo *Mt*PPAT (Morris and Izard, 2004; Timofeev V. I. et al., 2012; Timofeev V. et al., 2012), *Mt*PPAT in complex with substrates P-PantSH (**7**) and ATP (or its non-hydrolyzable analogue, AMPCPP) (Wubben and Mesecar, 2010; Timofeev V. et al., 2012), *Mt*PPAT in complex with the feedback inhibitor CoA (Timofeev et al., 2010; Wubben and Mesecar, 2011; Timofeev V. I. et al., 2012), and *Mt*PPAT in complex with reaction product DePCoA (Timofeev V. I. et al., 2012).

Structurally, *Mt*PPAT is part of the nucleotidyltransferase α/β phosphodiesterase superfamily, existing as a homohexamer, composed of six chemically identical subunits in its native state (Izard and Geerlof, 1999; Timofeev et al., 2010). Each subunit consists of 161 amino acid residues, with a five-stranded parallel β-sheet and six α-helices. The β-strands are linked by the α-helices in an alternating β/α pattern, resulting in the formation of a Rossman fold, the same nucleotide-folding pattern found in *Mt*PanC (Rao and Rossmann, 1973; Timofeev V. I. et al., 2012).

The active site of each subunit is comprised of a large cavity exposed to a solvent-filled channel; consequently, the entrance region of the channel is a functionally critical region of the enzyme that influences the rate of substrate access. A flexible loop consisting of residues 36–46 is thought to be responsible for restricting entrance to the active site. It contains Lys41, a conserved residue essential for stabilizing the pentavalent transition state of the reaction. Additionally, a group of negatively charged residues surround the entrance to the channel, specifically at the trimer–trimer interface, preventing the diffusion of negatively charged molecules from one trimer to another (Timofeev et al., 2010).

**Figure 8:**
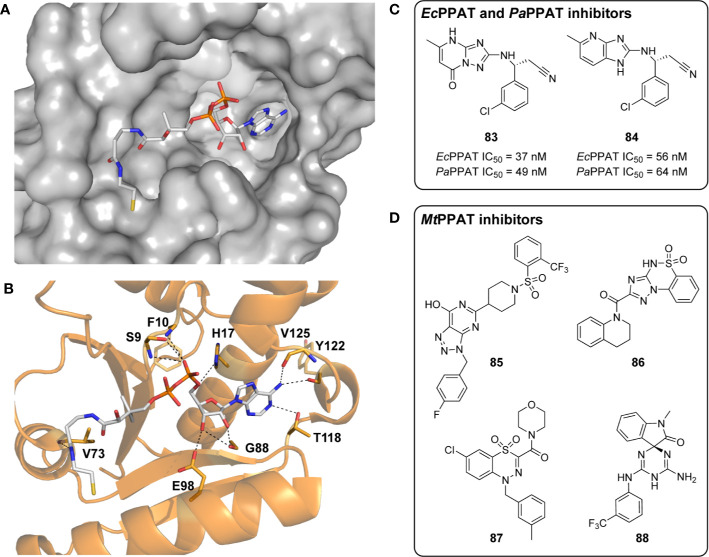
*Mt*PPAT structure and selected PPAT inhibitors. **(A)** *Mt*PPAT complexed with the reaction product DePCoA 12 shown as a stick structure (PDB: 3RBA) (Timofeev V. I. et al., 2012). **(B)** The binding interactions of DePCoA 12 in the active site of *Mt*PPAT (PDB: 3RBA). Hydrogen bonding interactions with the indicated active site residues are shown as black dashed lines. **(C)** Inhibitors of *E. coli* and *P. aeruginosa* PPAT displaying sub-micromolar activity (Moreau et al., 2018). **(D)** Selective inhibitors of *Mt*PPAT identified through *in silico* screening, which are predicted to prevent substrate binding at the active site (Podshivalov et al., 2017).

Previous studies have shown that in structures of *Mt*PPAT in complex with ATP and DePCoA (**12**), but not CoA (**1**) and P-PantSH (**7**), conformational changes occur in a small region of the polypeptide chain upon ligand binding. These rearrangements are mainly confined to residues 89–96, located at the perimeter of the solvent channel, which in turn cause alteration in the size of the solvent-filled channel and the overall quaternary structure (Timofeev V. et al., 2012). Interestingly, CoA controls the rate of the PPAT-catalyzed reaction through a feedback mechanism, in which CoA occupies the active site at high concentrations, causing the reaction to be terminated. Since the structures of CoA and DePCoA only differs in the presence of the additional 3′-phosphate, this raises the question as to how the structures are distinguished as to prevent product inhibition by DePCoA. Analysis of the two crystal structures in complex with *Mt*PPAT shows that the position of the pantetheine chain of both CoA and DePCoA are analogous; however, a significant distance between the amino groups of the adenylate group is observed for these two molecules (Timofeev et al., 2012). Additionally, DePCoA is located in a deep pocket of the active site of *Mt*PPAT (**Figure 8A**), unlike that of CoA. A detailed presentation of binding interactions of the enzyme with the reaction product, DePCoA, is shown in **Figure 8B**.

#### PPAT Inhibitors Developed Through Rational Design

While no inhibitors have been reported for *Mt*PPAT using structure-based approaches, inhibitors have been described for *Ec*PPAT, its homologue from *E. coli* (Zhao et al., 2003; Miller et al., 2010; Moreau et al., 2018; Skepper et al., 2018; Liyanage et al., 2019; Wang et al., 2020). The two homologues share 44% identity and 77% similarity in their amino acid sequences, and both exist as hexamers in their active form (Timofeev et al., 2010). While several differences are present between the two structures, such as the number of subunits involved in ligand binding, evaluation of *Ec*PPAT inhibitors can guide future design of compounds to inhibit *Mt*PPAT. Recently, a fragment-based strategy facilitated by crystallography revealed several highly potent compounds (**83** and **84**, **Figure 8C**) with nanomolar activity against *Ec*PPAT and *P. aeruginosa* PPAT (*Pa*PPAT), and moderate cellular activity against the efflux-deficient *E. coli* Δ*tolC* mutant (Moreau et al., 2018; Skepper et al., 2018). Inhibitor development studies of other bacterial PPAT enzymes, including those from *S. aureus, S. pneumoniae*, and *H. pylori* have also been performed, but none of these have led to the development of clinical candidates (De Jonge et al., 2013; Cheng et al., 2013). In the case of the *S. aureus* PPAT, potent enzyme inhibitors failed mainly due to poor drug-like properties (*e.g.*, low solubility and high clearance rates) that could not be improved without loss in potency (De Jonge et al., 2013).

#### PPAT Inhibitors Identified Through Screening

In a recent study, four compounds were identified as selective inhibitors of *Mt*PPAT found by virtual screening methods. Podshivalov et al. (2017) employed the Mcule online drug discovery platform to perform a docking-based virtual screen of more than four million compounds. The potency of lead compounds was analyzed based on the Vina scoring function (Lipinski et al., 2001; Trott and Olson, 2010). Four of the best-scoring compounds (**85**–**88**, **Figure 8D**), all of which satisfy Lipinski’s rules, were further investigated by molecular dynamics simulation to analyze their relative positions and binding interactions in the enzyme active site. Compounds **85**–**88** were all found to interact with residues in the active site of *Mt*PPAT, in addition to occupying a similar volume to that of native substrates (Podshivalov et al., 2017). Interestingly, binding of these ligands did not lead to any conformational change in active site residues of the enzyme. This differs to what is seen during binding of *Mt*PPAT’s substrates (ATP and DePCoA) and its feedback inhibitor CoA, all of which leads to significant conformational rearrangements. When docked to the enzyme’s active site, the compounds block substrate access to the active site, indicating that they would be able to act as inhibitors of *Mt*PPAT (Podshivalov et al., 2017). This study suggests that inhibitors of *Mt*PPAT could be designed as effective antimycobacterial agents with high selectivity.

#### Assessing PPAT as a Drug Target

The vast structural and mechanistic studies that have been reported for *Mt*PPAT demonstrate its potential as a target for antitubercular drug development. However, given the overall lack of inhibitors designed to date, there remains minimal knowledge about the potential efficacy of such compounds. Moreover, the targeted knockdown study conducted by Evans et al. (2016) indicated that *Mt*PPAT shows low vulnerability, as its depletion by transcriptional silencing could not reduce the protein to levels low enough to produce a growth phenotype. *Mt*PPAT is also not included in the list of targets identified by targetTB pipeline (Raman et al., 2008). Taken together, this would suggest that *Mt*PPAT is not a preferred target for antitubercular drug development at the moment.

### CoaE – Dephosphocoenzyme A Kinase (DPCK)

#### DPCK Enzyme Structure and Mechanism

DPCK, the final enzyme in the CoA biosynthetic pathway, catalyzes the ATP-dependent phosphorylation of DePCoA at the 3’-position of the ribose ring to generate CoA (**1**) (Strauss, 2010). Crystal structures of various bacterial DPCK proteins have been determined, yet the three-dimensional structure of Mtb DPCK (*Mt*DPCK), encoded by the *coaE* gene (Rv1631), remains unknown. Thus, homology modeling and sequence analysis using close structural homologues of *Mt*DPCK has been used in an attempt to elucidate the structure and function of this enzyme (Walia et al., 2009; Walia et al., 2011; Walia and Surolia, 2011).

DPCK enzymes belong to the NTP hydrolase superfamily, which is known to share several structural features even while only exhibiting minor sequence similarities (Walia et al., 2011). Most significant of these is the inclusion of a phosphate binding-loop, or P-loop, as well as the overall fold of the protein consisting of three domains. In the work of Walia et al. (2009), an exception to this prototype was discovered for *Mt*DPCK, in which an additional *C*-terminal domain that belongs to a family of proteins of unknown function, UPF0157, was identified and characterized. It was found that the UPF0157 domain plays an essential role in facilitating the expression and proper folding of the *N*-terminal domain, which was incapable of independently reaching its biologically functional conformation (Walia et al., 2009).

Biochemical and biophysical characterization of *Mt*DPCK was reported by Walia et al. (2009) following the construction of a mycobacterial DPCK homology model. The *N*-terminal domain was modelled after the structurally similar *E. coli* homologue (32.7% identity, 54.7% similarity), while the *E. faecalis* enzyme was used as a template to model the *C*-terminal domain (25.5% similarity). *Ef*DPCK also belongs to the UPF0157 family of proteins and no other crystal structures were available for this domain (Walia et al., 2009). This putative structure was then used to characterize the binding affinity of *Mt*DPCK to its substrates, DePCoA and ATP, and subsequently elucidate the order of substrate binding. The results revealed that *Mt*DPCK follows an ordered mechanism, beginning with the binding of DePCoA and followed by ATP, suggesting that a conformational change may occur upon binding of DePCoA that results in more favorable binding of ATP (Walia et al., 2009).

Binding and kinetic studies revealed that CTP binds tightly to *Mt*DPCK. Interestingly, when tested for its ability to act as a phosphate donor, CTP was unsuccessful in binding to the DePCoA–DPCK complex, but was found to inhibit *Mt*DPCK (Walia et al., 2009). This was explained through the docking of CTP and DePCoA to DPCK, which demonstrated the overlap of both molecules in the same binding pocket. The authors determined that CTP acts as a metabolic regulator. Since ATP is the phosphorylating agent, when the ratio of ATP/CTP in the cell is significantly reduced in response to stress, the CTP level effectively limits the amount of CoA produced (Walia et al., 2009).

#### DPCK Inhibitors Developed Through Rational Design or Screening

To date, there are no reported inhibitors of *Mt*DPCK. Due to the significant homology in both sequence and in structure between bacterial DPCKs and the DPCK domain of the bifunctional human coenzyme A synthase (CoASy) that has both PPAT and DPCK activity, the ability to develop selective inhibitors of the microbial DPCK enzymes may pose a significant challenge (Aghajanian and Worrall, 2002; Daugherty et al., 2002; Zhyvoloup et al., 2002; Moolman et al., 2014).

#### Assessing DPCK as a Drug Target

With no inhibitors of *Mt*DPCK being reported to date, the assessment of its suitability as a target mainly relies on the conditional knockdown mutant study of Evans et al. (2016) and the assessment of the targetTB target identification pipeline. Depletion of *Mt*DPCK through transcriptional silencing of *coaE* resulted in a progressive reduction of growth and a bacteriostatic phenotype, suggesting that inhibitors of *Mt*DPCK have a reasonable prospect of translating into whole cell growth inhibitors. This analysis is corroborated by the identification of *Mt*DPCK as one of 451 high confidence targets by targetTB (Raman et al., 2008). However, as pointed out above, the high sequence and structural homology between bacterial DPCK enzymes and the DPCK domain of eukaryotic CoASy proteins does suggest that selectivity might be difficult to achieve, which would in turn raise concerns regarding toxicity. In this context, it should be noted that the targetTB assessment does include a comprehensive comparative binding pocket analysis based on available structural information for the Mtb and human proteomes, and that *Mt*DPCK passed this specific screen (Raman et al., 2008). In addition, several other factors suggest that it could be possible to mitigate the potential challenge of selective inhibitor development. These include the possibility of exploiting the aspects related to the bifunctional nature of the human CoASy enzyme, and the fact that humans have a second, monofunctional DPCK which remains uncharacterized (Vozza et al., 2017; Giessner et al., 2018). This DPCK could provide redundancy that may counter any potential toxic effects from the use of DPCK-targeting inhibitors. Finally, it may be possible to target the regulation of *Mt*DPCK instead of its catalytic activity, to thereby circumvent any potential problems with selectivity.

## Conclusion

We have sought to provide a detailed overview of the current status of the Pan and CoA biosynthetic enzymes in the context of antiTB drug development. Although there is considerable support in the form of genetic and *in silico* validation studies of several of the pathway enzymes being high confidence drug targets, and although small molecule inhibitors with nanomolar potency has been developed in the case of some enzymes, unfortunately very few of these compounds show whole cell activity, with none being active *in vivo*. Yet, the information summarized here suggests that the CoA pathway remains a target with high potential for antiTB drug development. Specifically, *Mt*PanD and *Mt*CoaBC currently appear to be the most likely targets to yield positive results; the former based on the recent findings that link the activity of the well-known antiTB agent PZA (**52**) to inhibition and/or degradation of *Mt*PanD, and the latter based on the bacteriocidal effects and *in vivo* efficacy of targeted depletion of *Mt*CoaBC. *Mt*PanC also remains a worthy target based on the large number of leads for further development of improved inhibitors, while the inhibitory potential of *Mt*DPCK remains largely unexplored.

An important aspect of the development of inhibitors targeting the Mtb CoA pathway enzymes is that it should not be considered in isolation. Instead, such compounds are highly likely to be successful when used in combination with inhibitors of any number of enzymes that depend on CoA. For example, PptT inhibitors such as 8919 (**82**) (Ballinger et al., 2019), and inhibitors of enzymes involved in the glyoxylate shunt (such as isocitrate lyase, ICL) should both work synergistically with compounds reducing CoA levels (Wellington and Hung, 2018). This should also be the case for pantothenamides that are converted to CoA antimetabolites that target CoA utilizing enzymes (as seems to be the case in the malaria parasite, *Plasmodium falciparum*) (Schalkwijk et al., 2019). Considering that combination treatments have been the mainstay of antiTB drug regimens, multi-target inhibitors that all have a CoA producing or utilizing enzyme in the crosshairs could prove to be the breakthrough that is so urgently needed in the development of new antiTB drugs.

## Author Contributions

HSB, TJK, CSD, and ES designed the figures/tables and wrote the manuscript. HSB and TJK contributed equally. All authors contributed to the article and approved the submitted version.

## Funding

The authors gratefully acknowledge NIH R01AI136836 (to ES) for financial support, as well as financial support from the Stellenbosch University Open Access Publication Fund.

## Conflict of Interest

The authors declare that the research was conducted in the absence of any commercial or financial relationships that could be construed as a potential conflict of interest.
